# Maternal immunoglobulin G affects brain development of mouse offspring

**DOI:** 10.1186/s12974-024-03100-z

**Published:** 2024-05-02

**Authors:** Mizuki Sadakata, Kazuki Fujii, Ryosuke Kaneko, Emi Hosoya, Hisako Sugimoto, Reika Kawabata-Iwakawa, Tetsuhiro Kasamatsu, Shoko Hongo, Yumie Koshidaka, Akinori Takase, Takatoshi Iijima, Keizo Takao, Tetsushi Sadakata

**Affiliations:** 1 Education and Research Support Center, Gunma University Graduate School of Medicine, Maebashi, Gunma 371-8511 Japan; 2 Department of Behavioral Physiology, Faculty of Medicine, University of Toyama, Toyama, 930-0194 Japan; 3 Research Center for Idling Brain Science, University of Toyama, Toyama, 930-0194 Japan; 4 Life Science Research Center, University of Toyama, Toyama, 930-0194 Japan; 5 Medical Genetics Research Center, Nara Medical University, Kashihara, Nara 634-8521 Japan; 6 Division of Integrated Oncology Research, Gunma University Initiative for Advanced Research (GIAR), Gunma University, Maebashi, Gunma 371-8511 Japan; 7 Department of Medical Technology and Clinical Engineering, Gunma University of Health and Walfare, Maebashi, Gunma 371-0823 Japan; 8 Medical Science College Office, Tokai University, Isehara, Kanagawa 259-1193 Japan; 9 Department of Molecular Life Science, Division of Basic Medical Science and Molecular Medicine, School of Medicine, Tokai University, Isehara, Kanagawa 259-1193 Japan

**Keywords:** Microglia, Immunoglobulin G, Brain development

## Abstract

Maternal immunoglobulin (Ig)G is present in breast milk and has been shown to contribute to the development of the immune system in infants. In contrast, maternal IgG has no known effect on early childhood brain development. We found maternal IgG immunoreactivity in microglia, which are resident macrophages of the central nervous system of the pup brain, peaking at postnatal one week. Strong IgG immunoreactivity was observed in microglia in the corpus callosum and cerebellar white matter. IgG stimulation of primary cultured microglia activated the type I interferon feedback loop by Syk. Analysis of neonatal Fc receptor knockout (FcRn KO) mice that could not take up IgG from their mothers revealed abnormalities in the proliferation and/or survival of microglia, oligodendrocytes, and some types of interneurons. Moreover, FcRn KO mice also exhibited abnormalities in social behavior and lower locomotor activity in their home cages. Thus, changes in the mother-derived IgG levels affect brain development in offsprings.

**Supplementary Information:**

The online version contains supplementary material available at 10.1186/s12974-024-03100-z.

## Introduction

Microglia, originate as the first glial cells in the brain from the embryonic yolk sac [1, 2], migrate along the course of the fibers of the corpus callosum to all parts of the brain [3, 4]. Microglia act as the primary immune cell in the central nervous system (CNS), and form the first line of defense by regulating immunological and inflammatory responses [5]. Microglia exert an extensive array of physiological functions in the healthy brain, involving refinement of synaptic networks [6–9], promotion of developmental apoptosis [10], positioning of neurons within developing cortex [11], and secretion of growth factors for neuronal survival [12]. The activation of microglia has been implicated in the pathogenesis of various disorders, ranging from neuropsychiatric disorders (such as schizophrenia) to neurodevelopmental disorders (such as autism spectrum disorder (ASD)) [13, 14].

Breastfeeding is associated with long-term well-being, including low risks of infectious diseases and non-communicable diseases during childhood, such as asthma, cancer, autoimmune diseases, and obesity [15]. Breast milk is rich in microbiota and non-immune and immune components that ensure protection against numerous diseases and support the maturation of the developing immune system of an infant [16, 17]. On the other hand, an association between breastfeeding duration and adult intelligence has been reported [18, 19]. Long-term breastfeeding is also associated with fewer attention-deficit hyperactivity symptoms [20]. Moreover, social competence is related to breastfeeding duration [20]. However, the factors that affect brain development in breast milk are not well understood.

Immunoglobulins (Ig), such as IgG, IgA, and IgM, are present in breast milk [21]. We first discovered that maternal IgG binds specifically to microglia in some regions of the infant brain. Therefore, we examined how maternal IgG affects neonatal brain development by analyzing the brains of mouse offsprings lacking maternal IgG transfer. Downstream signal analysis using primary cultures and behavioral analysis were also performed to comprehensively analyze the effects of maternal IgG on brain development in the offspring.

## Materials and methods

### Clustered regularly interspaced palindromic repeats/ CRISPR-associated protein 9 -mediated deletion of the neonatal Fc receptor gene in vivo

Global FcRn (*Fcgrt*) KO mice were generated using CRISPR/Cas9 genome-editing technology in pronuclear stage embryos. The Cas9 protein, guide RNA, and single-stranded oligodeoxynucleotide (ssODN) were introduced into pronuclear stage embryos using the technique for animal knockout system by electroporation [22]. The Cas9 protein and guide RNA were purchased from Integrated DNA Technologies, Inc. (Coralville, IA, USA). Guide RNAs were designed to delete exons 2 and 3 of the *Fcgrt* gene of C57BL/6 mice (5’-AACAAGAGGCTGAGGGCCCA-3’ and 5’-GTGAGATGATACATCAGTGG-3’). Super electroporator NEPA21 (NEPA GENE Co. Ltd., Chiba, Japan) was used to introduce Cas9 protein, guide RNA, and ssODNs into embryos. On the following day, two-cell embryos were transferred into the oviduct ampulla (18–24 embryos per oviduct) of pseudopregnant ICR females (Japan SLC, Shizuoka, Japan). The mice were backcrossed to C57BL/6J mice (Japan SLC) for at least six generations, which was also expected to minimize genetic drift.

### Immunocytochemistry

Immunocytochemistry was performed as previously described [23]. Briefly, cells were fixed with Zamboni’s fixative (2% paraformaldehyde in 0.1 M phosphate buffer, pH 7.4, containing 0.2% picric acid) at room temperature (RT) for 15 min. After washing three times with phosphate-buffered saline (PBS), the cells were permeabilized in PBS containing 0.02% Triton X-100 at RT for 5 min. After blocking with Image-iT FX signal enhancer (Thermo Fisher Scientific, MA, USA) at RT for 60 min, the cells were incubated with primary antibodies at 4 °C overnight, rinsed in PBS, incubated with Alexa-conjugated secondary antibodies at RT for 1 h, and rinsed again in PBS. Immunoreactive cells were mounted in DAPI Fluoromount-G® mounting medium (SouthernBiotech, Birmingham, AL, USA) and observed under a fluorescence microscope (BX51; Olympus, Tokyo, Japan) equipped with a CCD camera (VB-7000; Keyence, Osaka, Japan). Researchers analyzing the images were blinded to the experimental conditions.

### Immunohistochemistry

Immunohistochemistry was performed as previously described [24]. Briefly, C57BL/6J male mice were deeply anesthetized with a combination of midazolam, medetomidine, and butorphanol tartrate and transcardially perfused with PBS, followed by Zamboni’s fixative. The tissues were dissected, post-fixed in Zamboni’s fixative at 4 °C for 5 h, and cryoprotected by immersion in 15% sucrose in PBS overnight at 4 °C. After embedding in Tissue-Tek OCT compound (Sakura Finetek, Tokyo, Japan), the tissues were frozen and sectioned at a thickness of 15 μm using a cryostat (CM1950, Leica Microsystems, Frankfurt, Germany) at − 18 °C. The sections were air-dried for 1 h and rinsed in PBS three times. After blocking with 5% bovine serum albumin (BSA) and 0.3% Triton X-100 in PBS at room temperature for 1 h, the sections were incubated at 4 °C overnight with primary antibodies in immunoreaction buffer (2×PBS containing 0.3% Triton X-100 and 1% BSA). The sections were then washed in PBS, incubated at room temperature for 1 h with the appropriate secondary antibodies in the immunoreaction buffer, and washed again in PBS. Stained sections were mounted in DAPI Fluoromount-G® mounting medium (SouthernBiotech) and observed by a fluorescence microscope (BX51; Olympus) equipped with a CCD camera (VB-7000; Keyence). Researchers analyzing the images were blinded to the experimental conditions.

### Immunoblot analysis

Cells were subjected to three washes with PBS and subsequently lysed using SDS sample buffer (50 mM Tris-HCl, pH 6.8, 2% SDS, 100 mM dithiothreitol, 10% glycerol, and 0.025% bromophenol blue). High-molecular-weight DNA was sheared by passing the lysate through a 29-gauge needle (326,666, BD Biosciences). After centrifugation at 15,000 rpm for 5 min and heat treatment at 55 °C for 20 min, the samples were electrophoresed. Detection was performed using horseradish peroxidase (HRP)-conjugated secondary mouse or rabbit antibodies and chemiluminescence HRP substrate (WBKLS0100, Millipore-Sigma), and the signals were captured using an image analyzer (LAS500, GE Healthcare, Chicago, IL, USA).

### Flow cytometry

To identify microglia using flow cytometry, previously described protocols were modified [25]. The neocortices, hippocampi, and corpus callosum of C57BL/6J mice were dissected, minced, and incubated with 500 U of type II collagenase (4176, Worthington Biochemical Corp, Freehold, NJ, USA) and 250 U of DNase (D4527, Millipore-Sigma, St. Louis, MO, USA) for 30 min at 37 °C in 10-ml Hanks’ Balanced Salt Solution (HBSS). The cells were triturated by repeated passage through 1-ml and 200-µL plastic micropipette tips every 5 min during incubation. After incubation, the cells were filtered through a 70-µm cell strainer (3-6649-02, ASONE, Osaka, Japan) and pelleted by centrifugation (400 × g, 5 min, 18 °C). Cell pellets were resuspended in 37% solution of isotonic Percoll (SIP) (17,089,102, Cytiva, Marlborough, MA, USA), overlaid with PBS, and centrifuged (200 × g for 40 min at 18 °C).

For the simultaneous identification of neurons, oligodendrocytes, and microglia using flow cytometry, established protocols were adapted and modified [25]. The neocortices, hippocampi, and corpus callosum of C57BL/6j mice were dissected, minced, and digested with 45 U papain (PAPL, Worthington), 0.1 kU/ml DNase I, 0.02% DL-cysteine, 0.02% bovine serum albumin (BSA), and 0.5% glucose in PBS at 200 rpm for 10 min at 37 °C. Fetal bovine serum (FBS) was added to a final concentration of 20%. The cells were triturated by repeated passage through 1-ml and 200-µL plastic micropipette tips, filtered through a 70-µm cell strainer (3-6649-02, ASONE), and pelleted in a centrifuge (300 × g, 3 min, 18 °C). Cell pellets were resuspended in 24% SIP (17,089,102, Cytiva, Marlborough, MA, USA), overlaid with PBS, and centrifuged (400 × g for 25 min at 18 °C).

After the centrifugation with SIP, erythrocytes were lysed using RBC lysing buffer (420,310, BioLegend), and 0.1% BSA–PBS containing 5 µg/ml of rat anti-CD16/32 antibody (C247, Leinco Technology, St. Louis, MO, USA) was used for blocking.

Intracellular antigens were stained using Fixation Buffer (420,801, BioLegend, San Diego, CA, USA) and Intracellular Staining Permeabilization Wash Buffer (421,002, BioLegend), according to the manufacturer’s instructions. Cell surface antigens were stained with unfixed cells before fixation.

Initial gating was performed on the live and singlet cells. Annexin V/7-AAD staining was performed using the PE Annexin V Apoptosis Detection Kit with 7-AAD (640,934, BioLegend), according to the manufacturer’s instructions. Staining was measured using a BD FACSCanto II flow cytometer (BD Biosciences, San Jose, CA, USA) and analyzed using FlowJo (BD Biosciences).

### Primary microglial culture

Primary microglia were isolated from the brains of newborn mice (P0), following previously described procedures [26]. Briefly, the hippocampi and neocortices were mechanically dissociated. The meninges were removed, and the remaining tissue was placed in a 25-ml HBSS with 20 U/ml DNase I and 0.01% trypsin and then incubated in a rocking rotor at 37 °C for 15 min. Thereafter, 0.01% soybean trypsin inhibitor (17,075,029, Thermo Fisher Scientific) was added, and the tissues were centrifuged at 400 × g for 5 min and dissociated via gentle trituration with a pipette in Dulbecco’s Modified Eagle Medium (DMEM) supplemented with 10% FBS. Single-cell suspensions from five brains were pooled in three poly L-lysine-coated cell cultivation flasks (2-8589-05; Violamo, Osaka, Japan) in DMEM supplemented with 10% FBS, 100 U/ml penicillin, and 100 µg/ml streptomycin (Thermo-Fisher Scientific). Subsequently, the medium was changed every four days. After 12 days of culture, the microglia were collected by shaking the flask at 200 rpm for 2 h. Detached microglia were then counted using a hemocytometer, plated at 4.5 × 10^5^ cells/ml in a poly L-lysine-coated 12-well plate, and placed in a 37 °C incubator. The primary microglia were maintained in these new plates for 3 days before treatment.

For microglial stimulation with IgG Fc, COS7 cells in 6-well plates were transfected with either pFUSE-mIgG1-Fc2, pFUSE-mIgG2A-Fc2, pFUSE-mIgG2B-Fc2, pFUSE-mIgG3-Fc2 (InvivoGen, San Diego, CA, USA), or an empty vector. Transfection was performed for 72 h using Lipofectamine 3000 reagent (Thermo-Fisher Scientific). The medium was collected and centrifuged at 400 × g for 5 min, and the supernatant was added to the primary cultured microglia. Whole-cell extracts of microglia stimulated for 4 h with the media were analyzed.

To measure the immunosignals of the IgG subclass using flow cytometry, primary cultured microglia stimulated for 2 h with 20 µg/ml of mouse IgG1, IgG2a, IgG2b, and IgG3 were trypsinized, and intracellular IgG immunosignals were analyzed using Fixation Buffer (420,801, BioLegend) and Intracellular Staining Permeabilization Wash Buffer (421,002, BioLegend), according to the manufacturer’s instructions.

To quantify the level of IFN-β in the supernatant, the culture media from IgG-stimulated microglia were collected and subjected to centrifugation at 400 × g for 5 min. The amount of IFN-β was measured using the LEGEND MAX Mouse IFN-β enzyme-linked immunosorbent assay Kit (439,407, BioLegend), according to the manufacturer’s instructions.

To measure the immunosignal intensity, the mean gray value of each cell was measured using ImageJ Fiji [27].

### RNA-sequencing analysis

RNA was extracted from microglia treated with PBS or IgG (10 µg/ml) at DIV15 for 24 h. After QC, mRNA was enriched using oligo(dT) beads, and rRNA was removed using the Ribo-Zero rRNA Removal Kit (Illumina, San Diego, CA, USA). First, the enriched mRNA was randomly fragmented using fragmentation buffer, followed by cDNA synthesis using the NEB Next Ultra RNA Library Kit (Illumina), according to the manufacturer’s protocols. The qualified libraries were pooled and fed into NovaSeq6000 sequencers (Illumina) according to the recommended pooling protocol based on the effective concentration and anticipated data volume. The filtering process for cleaning reads included the removal of reads containing adapters, those with *N* > 10% (where N represents an undetermined base), and those with low-quality bases (Q score ≤ 5), accounting for > 50% of the total bases. Clean reads were aligned with the mouse reference genome mm10 using hisat2 [28]. The *p*-value was calculated using the population stability index distribution for each group as the input. The *p*-value for this event was < 0.0001, considering the clear separation of the two distributions. In total, 373 DEGs (261 upregulated and 112 downregulated genes) with *p*-values < 0.01 and |logFC| > 0.5 were further analyzed using the Qiagen Ingenuity pathway analysis (IPA) software (version 81,348,237; Qiagen, Hilden, Germany).

### Behavioral tests

Animals were cared for and treated according to the ‘Japanese Act on the Welfare and Management of Animals’ and the ‘Guidelines for the Proper Conduct of Animal Experiments’ issued by the Science Council of Japan. All experimental protocols were reviewed and approved by the Gunma University Animal Care and Experimentation Committee. Unless otherwise noted, most behavioral tests were performed as previously described [29]. Male mice older than 11 weeks were subjected to different behavioral tests. Mice were housed two to four per cage (one to three for each genotype) in a room with a 12-hour light/dark cycle (lights on at 7:00 am), with access to food and water ad libitum. Behavioral tests were performed between 9:00 am and 6:00 pm. After each test, the testing apparatus was cleaned with super hypochlorous water to prevent bias due to olfactory cues unless otherwise noted.

#### Neuromuscular strength test

A grip strength meter (O’HARA & Co., Tokyo, Japan) was used to assess the forelimb grip strength. The peak force applied by the forelimbs of the mouse was recorded in Newtons (N). Each mouse was tested three times, and the highest value was used for data analysis. In the wire hang test, the mouse was placed on a wire mesh that was then slowly inverted so that it gripped the wire to prevent it from falling off. Latency to fall was recorded, with a 60 s cutoff time.

#### Rotarod test

Motor coordination and balance were assessed using the rotarod test. This test, which uses an accelerating rotarod (UGO Basile, Varese, Italy), was performed by placing mice on rotating drums (3 cm diameter) made of polyvinyl chloride (PVC) and measuring the time each animal was able to maintain its balance on the rod. The speed of the rotarod was accelerated from 4 to 40 rpm over a 5-minute period. All mice were subjected to the test without pre-test training.

#### Hot plate test

The hot plate test was used to evaluate the sensitivity to a painful stimulus. Mice were placed on a 55 °C hot plate with a black anodized aluminum floor (Columbus Instruments, Columbus, OH, USA), and the latency to the first fore- or hind paw response was recorded with a 15 s cutoff time. The paw response was defined as either a paw lick or foot shake.

#### Open field test

Each mouse was placed in the corner of an open field apparatus (40 × 40 × 30 cm; Accuscan Instruments, Columbus, OH, USA), which consisted of a white plastic floor and transparent Plexiglas wall. The total distance traveled (cm), vertical activity, time spent in the center area (20 cm × 20 cm), and beam-break counts for stereotyped behaviors were recorded. Immediately after the mice were placed in the arena, their behavior was recorded for 120 min.

#### Social interaction test in a novel environment

In the social interaction test, two mice of identical genotypes that were previously housed in different cages were placed together in a white PVC plastic box (40 × 40 × 30 cm) (O’HARA & Co.) and allowed to explore freely for 10 min. Behavior was recorded and analyzed automatically using Image SI software (see Section, “Data analysis”). The total number of contacts, total duration of active contact, total contact duration, mean duration per contact, and total distance traveled were measured. If the two mice contacted each other and the distance traveled by either mouse was longer than 10 cm, the behavior was classified as an “active contact.”

#### Crawley’s sociability and social novelty preference test

This test is a well-designed method for investigating the effects of complex genetics on sociability and preference for social novelty [30]. The testing apparatus consisted of a rectangular, three-chambered PVC plastic box and lid with an infrared video camera (O’HARA & Co.). We modified the method as follows: a habituation session was performed in the apparatus for 10 min the day before the sociability test, and the wire cages in the lateral compartments were located in the corners of each compartment. In the sociability test, an unfamiliar C57BL/6J male mouse (stranger) that had no prior contact with the subject mouse was placed in one of the side chambers. The subject mouse was first placed in the middle chamber and allowed to explore the entire test box for a 10-minute session. In the social novelty preference test, each mouse was tested in a 10-minute session to quantify social preference for a new stranger. After the first 10-minute session, a second unfamiliar mouse was placed in the chamber that had been empty during the first 10-minute session. Data acquisition and analysis were performed automatically using the Time CSI software (O’Hara & Co.).

#### Locomotor activity monitoring in home cage

Locomotor activity monitoring in the home cage was performed using a system that automatically analyzed the locomotor activity of mice in their home cage. The system contained a home cage (29 × 18 × 12 cm), a filtered cage top, and an infrared video camera attached to the top of a stand. Each mouse was housed individually in a home cage. Five hours after the mice were placed in their home cages, the light was turned off, and the analysis was performed. Images from each cage were captured at a rate of one frame per second, and the distance traveled was measured automatically using Image HA software (see Section, “Data analysis”).

#### Startle response/prepulse inhibition (PPI) test

A startle reflex measurement system (O’HARA & Co.) was used to measure acoustic startle response and PPI. The test session began by placing a mouse in a transparent PVC plastic cylinder, where it was left undisturbed for 10 min. White noise (40 ms) was used as the startle stimulus for all the trial types. Startle response was recorded for 400 ms starting with the onset of the startle stimulus. The background noise level in each chamber was 70 dB. The intensity of the startle stimulus was 110 dB or 120 dB. The prepulse sound was presented 100 ms before the startle stimulus, and its intensity was 74 dB or 78 dB. Four combinations of prepulse and startle stimuli were used. Six blocks of the six trial types were presented in a pseudo-random order such that each trial type was presented once within a block.

#### Data analysis

Behavioral data were obtained automatically through applications based on ImageJ software and were modified for each test by Tsuyoshi Miyakawa (available through O’HARA & Co.). The ImageJ plugins, and the precompiled plugins for open field test (Image OF) and home cage test (Image HA) are freely available on the website of “Mouse Phenotype Database” (http://www.mouse-phenotype.org/software.html).

### Statistical analyses

Statistical analyses were performed using Excel Statistics (Statcel 3; Social Survey Research Information, Tokyo, Japan). All data are presented as the mean ± SEM, unless otherwise noted. Differences between groups were analyzed using the Student’s *t*-test, Mann-Whitney U test, one-way analysis of variance (ANOVA), two-way repeated measures ANOVA, and chi-squared test according to each experimental design.

**Table:** **Key resources table**

Reagent type (species) or resource	Designation	Source or reference	Identifiers	Additional information
antibody	anti-Stat1 (pY701) (Mouse monoclonal)	BD Biosciences	Cat# 612,232	WB (1:500)
antibody	anti-Stat1 (Mouse monoclonal)	BD Biosciences	Cat# 610,115	WB (1:500)
antibody	anti-actin (Rabbit polyclonal)	Millipore-Sigma	Cat# A5060	WB (1:1000)
antibody	anti-Syk (pY525/526) (Rabbit polyclonal)	Cell Signaling Technology	Cat# 2711	WB (1:1000)
antibody	anti-Iba1 (Rabbit polyclonal)	Wako Pure Chemical	Cat# 019-19741	IF (1:1000)
antibody	anti-CD16/32 (Rat monoclonal)	Leinco Technologies	Cat# C247	IF (1:250)
antibody	anti-CD11b (Rat monoclonal)	Abcam	Cat# ab6332	IF (1:500)
antibody	anti-IgA (Rat monoclonal)	SouthernBiotech	Cat# 1165-01	IF (1:250)
antibody	anti-MBP (Mouse monoclonal)	BioLegend	Cat# SMI-99P	IF (1:500)
antibody	anti-MAG (Mouse monoclonal)	Millipore-Sigma	Cat# MAB1567	IF (1:200)
antibody	anti-VIP (Rabbit polyclonal)	OriGene	Cat# BP882	IF (1:500)
antibody	anti-calbindin (Rabbit polyclonal)	Millipore-Sigma	Cat# AB1778	IF (1:1000)
antibody	anti-parvalbumin (Mouse monoclonal)	Millipore-Sigma	Cat# P3088	IF (1:500)
antibody	anti-VGAT (Rabbit polyclonal)	Synaptic Systems	Cat# 131,002	IF (1:1000)
antibody	anti-GAD67 (Mouse monoclonal)	Millipore-Sigma	Cat# MAB5406	IF (1:500)
antibody	anti-cleaved caspase-3 (Rabbit monoclonal)	Cell Signaling Technology	Cat# 9664	IF (1:1000)
antibody	anti-Ki67 (Rat monoclonal)	BioLegend	Cat# 652,401	IF (1:200)
antibody	anti-Rabbit IgG H&L (Alexa Fluor® 488) (Goat polyclonal)	Thermo Ficher Scientific	Cat# A31628	IF (1:1000)
antibody	anti-Mouse IgG H&L (Alexa Fluor® 555) (Donkey polyclonal)	Abcam	Cat# ab150110	IF (1:1000)
antibody	anti-Rat IgG H&L (Alexa Fluor® 555) (Goat polyclonal)	Thermo Ficher Scientific	Cat# A21434	IF (1:1000)
antibody	anti-Rat IgG H&L (Alexa Fluor® 488) (Goat polyclonal)	Thermo Ficher Scientific	Cat# A11006	IF (1:1000)
antibody	anti-Rabbit IgG H&L (Alexa Fluor® 555) (Goat polyclonal)	Thermo Ficher Scientific	Cat# A31630	IF (1:1000)
antibody	anti-Mouse IgG H&L (Alexa Fluor® 488) (Goat polyclonal)	Thermo Ficher Scientific	Cat# A31620	IF (1:1000)
antibody	anti-CD11b (Alexa Fluor® 488) (Rat monoclonal)	BioLegend	Cat# 103,131	FC (1:200)
antibody	anti-CD45 (PerCP/Cy5.5) (Rat monoclonal)	BioLegend	Cat# 101,217	FC (1:200)
antibody	anti-CD45 (APC/Cy7) (Rat monoclonal)	BioLegend	Cat# 103,115	FC (1:200)
antibody	anti-mouse IgG (APC) (Goat polyclonal)	BioLegend	Cat# 405,308	FC (1:100)
antibody	anti-IgA (biotin) (Rat monoclonal)	BioLegend	Cat# 407,003	FC (1:100)
antibody	anti-CD200 (PE/Cy7) (Rat monoclonal)	BioLegend	Cat# 123,817	FC (1:200)
antibody	anti-CD31 (APC) (Rat monoclonal)	BioLegend	Cat# 102,509	FC (1:200)
antibody	anti-O4 (APC) (Mouse monoclonal)	Miltenyi Biotec	Cat# 130-119-155	FC (1:400)
antibody	anti-CD16 (PE) (Rat monoclonal)	BioLegend	Cat# 158,003	FC (1:200)
antibody	anti-CD32 (PE) (Rat monoclonal)	BioLegend	Cat# 156,403	FC (1:100)
antibody	anti-CD64 (PE) (Rat monoclonal)	BioLegend	Cat# 139,303	FC (1:100)
antibody	IgG2a (PE) (Rat monoclonal)	BioLegend	Cat# 400,507	Isotype control
antibody	IgG2b (PE) (Rat monoclonal)	BioLegend	Cat# 400,607	Isotype control
antibody	IgG1 (PE) (Mouse monoclonal)	BioLegend	Cat# 400,111	Isotype control
antibody	IgG (APC) (Goat polyclonal)	SouthernBiotech	Cat# 0109 − 11	Isotype control
antibody	IgG1 (biotin) (Rat monoclonal)	BD Biosciences	Cat# 553,923	Isotype control
cell line	HMC3	ATCC	Cat# CRL-3304	Human microglial cell line
recombinant protein	IFN-β1	BioLegend	Cat# 581,302	3 pg/ml
chemical compound	Ruxolitinib	Cayman Chemical	Cat# 11,609	1–5 µM
chemical compound	PRT062607	Selleck Chemicals	Cat# S8032	1 µM
chemical compound	BAY61-3606	Millipore-Sigma	Cat# 574,714	5–10 µM
antibody	anti-IFNAR1 (Mouse monoclonal)	BioLegend	Cat# 127,302	Blocking (2 µg/ml)
antibody	IgG from mouse serum	Millipore-Sigma	Cat# I5381	1–100 µg/ml
antibody	Mouse IgG1	BioLegend	Cat# 401,401	20 µg/ml
antibody	Mouse IgG2a	BioLegend	Cat# 401,501	20 µg/ml
antibody	Mouse IgG2b	BioLegend	Cat# 402,201	20 µg/ml
antibody	Mouse IgG3	BioLegend	Cat# 401,301	20 µg/ml
antibody	Mouse IgM	Thermo Ficher Scientific	Cat# 14-4752-82	10 µg/ml
antibody	Mouse IgE	BioLegend	Cat# 401,701	10 µg/ml
antibody	Mouse IgA	Thermo Ficher Scientific	Cat# 14-4762-81	10 µg/ml
antibody	Human IgG	Millipore-Sigma	Cat# I4506	100 µg/ml

## Results

### IgG immunoreactivity in the corpus callosum and cerebellar white matter microglia of the mouse infant brain

**Fig. 1:**
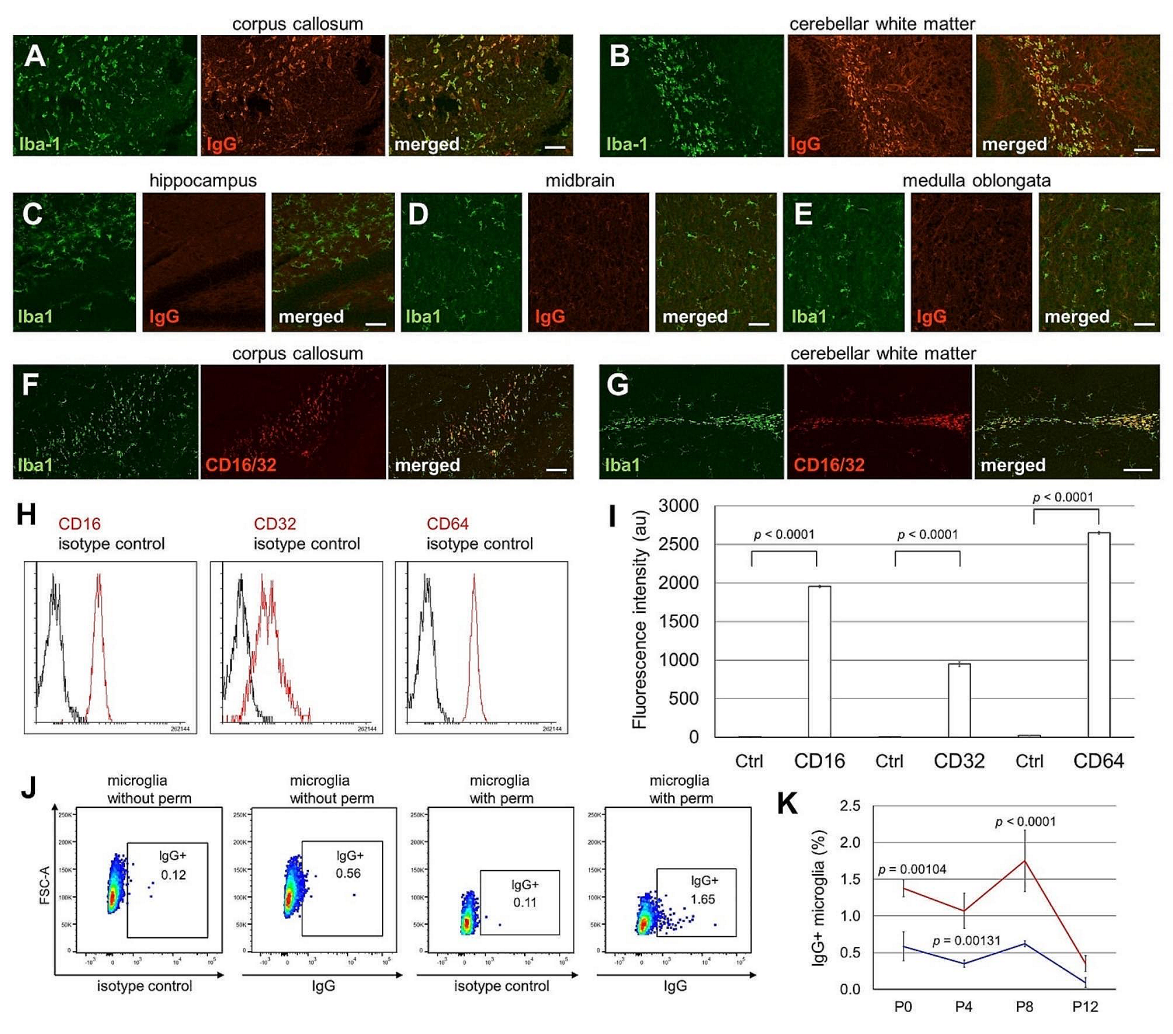
Immunoreactivity of IgG in microglia of the brains of infant mice. (**A–E**) Sagittal sections of C57BL/6J mice at postnatal day 8 (P8) were immunolabeled with an anti-Iba1 antibody (green) and anti-mouse IgG antibody (red). Scale bars, 50 μm. (**F, G**) Sagittal sections of mice at P8 were immunolabeled with anti-Iba1 antibody (green) and anti-CD16/32 antibody (red). Scale bars, 100 μm. (**H**) Flow cytometry data showing staining of microglia with specific antibodies (red) and isotype control (black) at P8. (**I**) Flow cytometry quantification of the expression of CD16, CD32, and CD64 in microglia is indicated by fluorescence intensity. Ctrl, isotype control. The *p*-values from Student’s *t*-test are indicated. (**J**) Representative plots of flow cytometry data showing staining of microglia with anti-mouse IgG antibodies and isotype control at P8, with or without fixation and permeabilization. (**K**) Quantification of the percentage of microglia stained with anti-mouse IgG, with (red) or without (blue) fixation and permeabilization, at P0, P4, P8, and P12 [P0 with permeabilization (*n* = 3); P0 without permeabilization (*n* = 3); P4 with permeabilization (*n* = 4); P4 without permeabilization (*n* = 3); P8 with permeabilization (*n* = 7); P8 without permeabilization (*n* = 3); P12 with permeabilization (*n* = 3); P12 without permeabilization (*n* = 3)]. Two-way analysis of variance revealed a significant effect on localization (*p* < 0.0001) and stage (*p* < 0.0001). *P*-values from Fisher’s protected least-significant difference (PLSD) post hoc tests for pairwise comparisons are indicated

We found that microglia exhibited IgG immunoreactivity in the mouse corpus callosum (Fig. 1A) and cerebellar white matter (Fig. 1B) on postnatal day 8 (P8). Immunoreactivity for IgG was either weak or absent in the hippocampus (Fig. 1C), midbrain (Fig. 1D), and medulla oblongata (Fig. 1E).

IgG Fc receptors fall into three classes– CD16 (FcγRIII), CD32 (FcγRII) and CD64 (FcγRI). CD16 (FcγRIII) and CD32 (FcγRII) are low-to-medium affinity receptors and mediate signaling after cross-linking of the receptors on the cell surface, whereas CD64 (FcγRI) is a high affinity receptor for IgG [31]. Immunoreactivity of the antibody that recognizes both CD16 and CD32 was observed in the microglia of the corpus callosum (Fig. 1F) and cerebellar white matter (Fig. 1G). We also examined the expression of the three IgG receptor classes using flow cytometry. Microglia in the telencephalon were analyzed (see Materials and Methods). The results showed that all three IgG receptor classes were expressed in microglia (Fig. 1H and I).

Border-associated macrophages (BAMs) reside at the interface between the brain and periphery, including the meninges and choroid plexus [32]. Immunoreactivity for IgG was also observed in BAMs at the choroid plexus (Fig. S1A) and pia mater (Fig. S1B). These BAMs also showed immunoreactivity for CD16/32 (Fig. S1C and S1D).

Next, intracellular and cell surface IgG localizations were examined using flow cytometry. The percentage of microglia showing IgG immunoreactivity was higher when the cells were permeabilized than that without permeabilization at P8 (Fig. 1J). The percentage of microglia showing IgG immunoreactivity peaked at P8 and was almost nonexistent at P12 (Fig. 1K). Accordingly, at P15, the immunoreactivity for IgG in microglia in the corpus callosum (Fig. S2A) and cerebellar white matter (Fig. S2D) was very weak. At P28 and P56, little immunoreactivity for IgG in the microglia in the corpus callosum (Fig. S2B and S2C) and cerebellar white matter (Fig. S2E and S2F) was observed. In contrast, immunoreactivity for IgG in BAMs was higher at P28 (Fig. S2H) than at P15 (Fig. S2G) and P56 (Fig. S2I). Similarly, results for the pia mater were obtained (Fig. S2J,S2K, and S2L).

The corpus callosum and cerebellar white matter of the infant brain, called the “fountain of microglia,” shows large amounts of amoeboid microglia and high expression of Mac3 up to P10 [33]. Immunoreactivity for IgG in microglia in the corpus callosum was heterogeneous and particularly strong in the “fountain of microglia,” where cells were densely packed when observed using 4’,6-diamidino-2-phenylindole (DAPI) (Fig. S3A). CD11b immunoreactivity in microglia in the corpus callosum was also heterogeneous (Fig. S3B), and microglia with strong IgG immunoreactivity were observed in parallel with microglia with strong CD11b immunoreactivity (Fig. S3C). When the expression levels of CD11b and FcγR were examined by flow cytometry, the expression of CD16, CD32, and CD64 was positively correlated with that of CD11b (Fig. S3D, S3E and S3F).

### Activation of type I interferon-related genes in primary cultured microglia by IgG

**Fig. 2:**
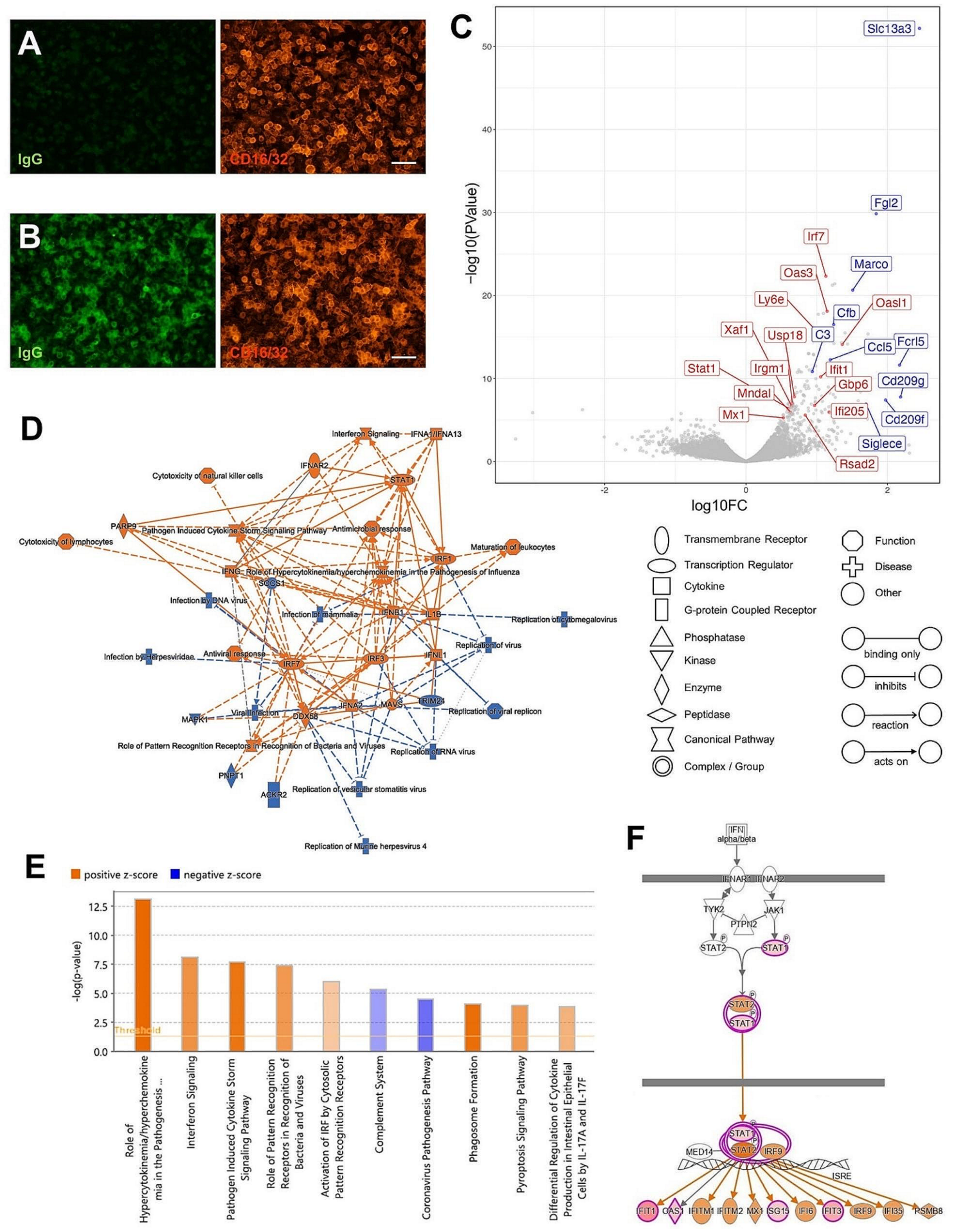
Comprehensive analysis of microglial genes differentially expressed by IgG stimulation. (**A, B**) Primary cultured microglia were immunolabeled with anti-mouse IgG antibody (green) and anti-CD16/32 antibody (red) without IgG addition (**A**) and 24 h after IgG addition to the culture media (**B**). Scale bars, 50 μm. (**C**) Volcano plots of DEGs between the control and primary cultured microglia stimulated with 10 µg/ml IgG for 24 h. Representative IFN-I-related genes are shown in red. (**D**) Graphical summary of the pathways regulated after IgG treatment generated by ingenuity pathway analysis (IPA). Orange and blue indicate activation and inhibition, respectively. The solid line indicates a direct interaction, the dashed line indicates an indirect interaction, and the dotted line indicates an inferred correlation from machine-based learning. (**E**) Significant pathways identified by IPA. Positive and negative z-scores, colored in orange and blue, represent the activation and inhibition of pathways, respectively. (**F**) Significant activation of the IFN-I signaling pathway was predicted using IPA. Orange indicates activation

To examine the effects of IgG binding on microglia, we prepared primary microglia cultures. Primary cultured microglia showed CD16/32 (Fig. 2A) and IgG immunoreactivity after the addition of IgG to the culture medium (Fig. 2B). Immunostaining for IgG in microglia was strongly observed up to 24 h after addition to the medium; however, degradation was observed after 48 h (Fig. S4A and S4B).

We identified differentially expressed genes (DEGs) between the control and IgG-treated primary cultured microglia based on RNA sequencing (RNA-seq) data. Functional annotation analysis showed significant enrichment of genes involved in the response to type I interferon (IFN-I), IFN-α, and IFN-β and the regulation of IFN-I production (Table S1). Volcano plots display the statistical significance of the difference relative to the magnitude of difference for each gene in the comparison. In the volcano plots, IFN-I-related gene expression increased in response to IgG stimulation (Fig. 2C). Figure 2D shows the clusters generated by pathway analysis of DEGs between control and IgG-stimulated microglia. IFN-I genes (i.e., IFNA1, IFNA2, and IFNB1), receptor of IFN-I (IFNAR2), and interferon regulatory factors (i.e., IRF1, IRF3, and IRF7) were included in this gene network.

**Fig. 3:**
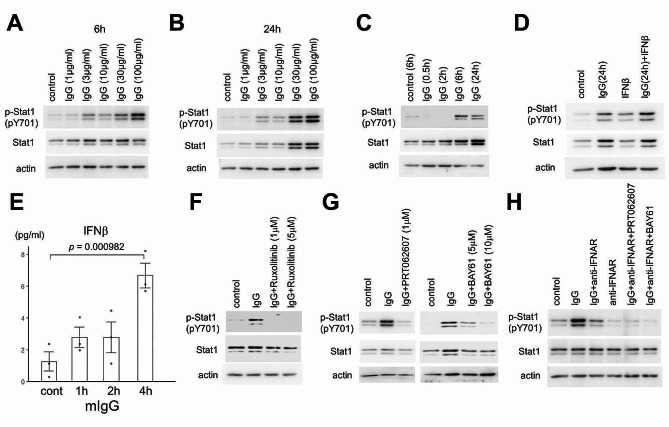
IgG activates the IFN-I feedback loop via Syk. (**A**) Immunoblot analysis of primary cultures of IgG-stimulated microglia. Protein lysates were immunoblotted using anti-phosphorylated Stat1 (pY701), anti-Stat1, and anti-actin antibodies. Whole-cell extracts of microglia stimulated for 6 h with mouse IgG were analyzed. (**B**) Whole-cell extracts of microglia stimulated for 24 h with mouse IgG were analyzed. (**C**) Whole-cell extracts of microglia stimulated with 10 µg/ml mouse IgG were analyzed. (**D**) Whole-cell extracts of microglia stimulated with 10 µg/ml mouse IgG for 24 h and 3 pg/ml IFN-β for 30 min were analyzed. (**E**) IFN-β secreted from primary cultured microglia in response to 10 µg/ml mouse IgG stimulation was measured using enzyme-linked immunosorbent assay (ELISA) (*n* = 3 each). One-way analysis of variance showed a significant difference (*p* = 0.00545). The *p*-values from Fisher’s PLSD post hoc tests are indicated. (**F**) Whole-cell extracts of microglia stimulated with 10 µg/ml mouse IgG with 1 or 5 µM ruxolitinib for 6 h were analyzed. (**G**) Whole-cell extracts of microglia stimulated with 10 µg/ml mouse IgG for 6 h with 1 µM PRT062607 and 5 or 10 µM BAY61-36061 were analyzed. PRT062607 and BAY61-36061 were added to the medium 1 h before the addition of IgG. (**H**) Whole-cell extracts of microglia stimulated with 20 µg/ml mouse IgG for 6 h with 2 µg/ml anti-IFNAR, 1 µM PRT062607, or 10 µM BAY61-3606 were analyzed. PRT062607 and BAY61-36061 were added to the medium 1 h before the addition of IgG

Ingenuity pathway analysis of DEGs revealed altered canonical pathways that were either activated or inhibited (Fig. 2E). Cytokine storm signaling and phagosome formation pathways were also activated, in addition to the interferon signaling pathway (Fig. 2E). The IFN-I signaling pathway and upregulated genes are shown in Fig. 2F. Stat1 and Stat2 are key markers of IFN-I activation and transcription of interferon-stimulated gene (ISG) expression [34]. Binding of IFN-I to its receptors initiates a signaling cascade that results in the phosphorylation and heterodimerization of Stat1 and Stat2 to form the active transcription factor complex. Therefore, we examined whether Stat1 was phosphorylated in IgG-stimulated microglial cells. We found that, in primary cultured microglia, Stat1 was phosphorylated in a dose-dependent manner by 6 h IgG stimulation (Fig. 3A). IgG concentrations in mouse blood are generally in the range of 1 to 10 mg/ml [35–37]. Here, we found that Stat1 was phosphorylated at much lower concentrations as shown in Fig. 3A. IgG stimulation for 24 h similarly phosphorylated Stat1 in a dose-dependent manner (Fig. 3B). However, the Stat1 protein also increased in a dose-dependent manner, indicating that phosphorylated Stat1 increased proportionally (Fig. 3B). Long-term stimulation with interferon increases Stat1 protein levels and induces a larger response to subsequent stimulation by interferon, a phenomenon termed priming [38]. As shown in Fig. 3C, IgG-stimulated phosphorylation of Stat1 began after 6 h, and Stat1 protein levels also increased in a time-dependent manner. When IFN-β was added 24 h after IgG treatment, Stat1 phosphorylation was elevated compared with that after the addition of IFN-β alone, depending on the increase in the Stat1 protein (Fig. 3D).

In Fig. 3E, IgG stimulation induced IFN-β secretion from primary cultured microglia. IFN-I production can be amplified using a positive feedback loop (Fig. S5). All IFN-I signals through the IFN-I receptors IFNAR1 and IFNAR2 dimerize upon ligand binding and activate Jak1 and Tyk2 receptor tyrosine kinases (Fig. S5). Activated Jak1 phosphorylates the intracellular domain of the receptor and facilitates the recruitment and phosphorylation of Stat [39]. Ruxolitinib, which is a selective inhibitor of Jak1 and Jak2, inhibited the phosphorylation of Stat1 in IgG-stimulated microglia (Fig. 3F).

How does IgG stimulation activate the IFN-I feedback loop? Syk is essential for signaling through immune receptors involved in the recognition of antigens, immunoglobulins, and cell surface proteins (Fig. S5) [40]. Reports have shown that Syk is associated with the priming of interferon signaling by phosphorylating the Stat1 protein [41]. Syk inhibitors—PRT062607 and BAY61-3606— strongly suppressed the Stat1 phosphorylation in IgG-stimulated microglia (Fig. 3G).

When feedback was inhibited by the IFN-I blocker anti-IFNAR alone, Stat1 phosphorylation in response to IgG stimulation was only partially reduced (lane 3, Fig. 3H). Co-treatment with Syk inhibitors (PRT062607, BAY61-3606) and anti-IFNAR further inhibited the Stat1 phosphorylation in response to IgG stimulation (lanes 5 and 6, Fig. 3H), indicating that Syk is upstream of the IFN-I feedback loop (Fig. S5).

**Fig. 4:**
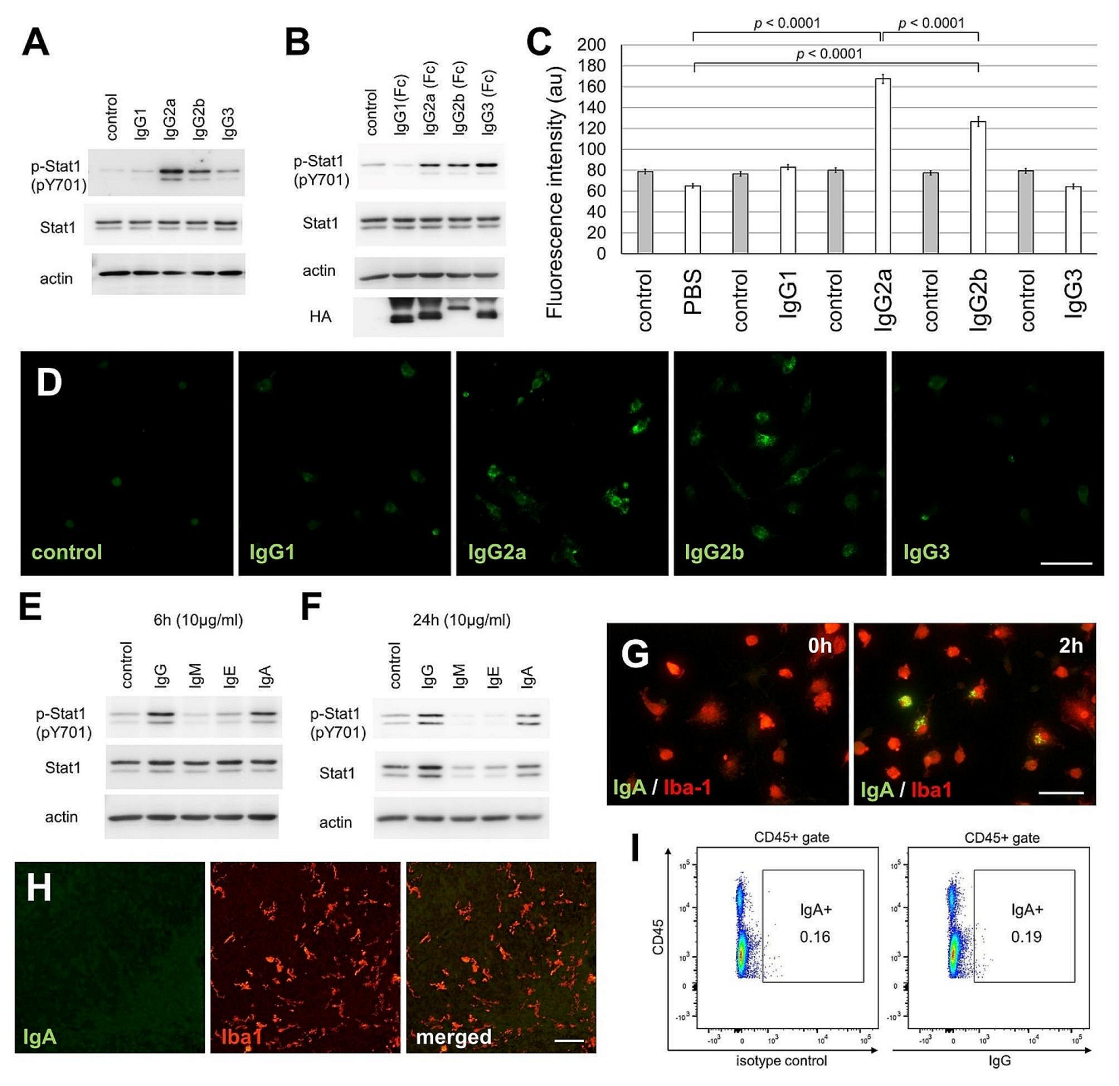
Differences in the IgG subclass and immunoglobulin class for Stat1 phosphorylation. (**A**) Immunoblot analysis of primary cultures of IgG-stimulated microglia. Whole-cell extracts of microglia stimulated with 20 µg/ml mouse IgG for 4 h were analyzed. (**B**) Whole-cell extracts of microglia stimulated with mouse IgG Fc secreted from COS7 cells for 4 h were analyzed (see Materials and Methods). (**C**) Flow cytometry quantification of IgG subclass immunosignals in microglia is indicated using fluorescence intensity. Primary microglia culture were incubated for 2 h with Mouse IgG1, IgG2a, IgG2b, and IgG3 (20 µg/ml) [IgG3 (*n* = 369); others (*n* = 494)]. A two-way analysis of variance revealed a significant effect of IgG stimulation (*p* < 0.0001) and IgG subclass (*p* < 0.0001). The *p*-values from Fisher’s partial least significant difference (PLSD) post hoc tests are indicated. (**D**) Primary cultured microglia were immunolabeled with anti-mouse IgG antibody (green). Mouse IgG1, IgG2a, IgG2b, and IgG3 (20 µg/ml) were added to the culture media of primary cultured microglia 2 h before fixation. Scale bar, 40 μm. (**E, F**) Immunoblot analysis of primary cultures of microglia stimulated with immunoglobulin. Whole-cell extracts of microglia stimulated for 6 h (**E**) and 24 h (**F**) with 10 µg/ml mouse IgG, IgM, IgE, and IgA (monomer) were analyzed. (**G**) Primary cultured microglia were immunolabeled with anti-mouse IgA (green) and anti-Iba1 (red) antibodies. Images were capture before IgA addition and 2 h after the addition of 10 µg/ml IgA to the culture media. Scale bar, 40 μm. (**H**) Sagittal sections of C57BL/6J mice corpus callosum at P8 were immunolabeled with anti-IgA (green) and anti-Iba1 (red) antibodies. Scale bars, 50 μm. (**I**) Flow cytometry data showing staining of all CD45-positive cells, including microglia, with anti-CD45 and anti-mouse IgA antibodies or an isotype control at P8 after fixation and permeabilization

Mouse IgG can be divided into four subclasses: IgG1, IgG2a, IgG2b, and IgG3, with IgG2c being the equivalent of IgG2a in some mouse strains [42]. In primary cultures of microglia prepared from C57BL/6J mice, Stat1 was phosphorylated by IgG2a and IgG2b stimulation (Fig. 4A). Furthermore, the Stat1 protein was phosphorylated only by stimulating the Fc (constant) region of IgG2a, IgG2b, and IgG3 (Fig. 4B). Flow cytometry results showed that the binding activity of IgG2a was higher than that of IgG2b (Fig. 4C). Immunocytochemistry also revealed the binding of IgG2a and IgG2b to primary cultured microglia (Fig. 4D).

When the classes of immunoglobulins other than IgG, IgM, IgE, and IgA were examined, stimulation with monomeric IgA also phosphorylated Stat1 (Fig. 4E and F). When IgA was added to the culture media, IgA binding was observed in a few microglia (Fig. 4G). However, IgA immunoreactivity was not observed in the brains of infant mice (Fig. 4H). Furthermore, IgA immunoreactivity was not observed in all CD45-positive cells, including microglia, in the infant mouse brain using flow cytometry (Fig. 4I).

A study reported that some mouse strains show IgG immunoreactivity in epithelial cells and microglia, whereas others, such as CBA/J and CBA/N mice [43], do not show IgG immunoreactivity. However, IgG immunoreactivity was observed in the brains of CBA/N mice (Fig. S6A). Upon stimulation with IgG, Stat1 was phosphorylated in primary cultured microglia prepared from CBA/N mice (Fig. S6B). Moreover, both the Syk and Stat1 proteins were phosphorylated by stimulation with human IgG in the human microglial HMC3 cell line (Fig. S6C).

### Effects of lack of maternal IgG transfer on offspring brain development

**Fig. 5:**
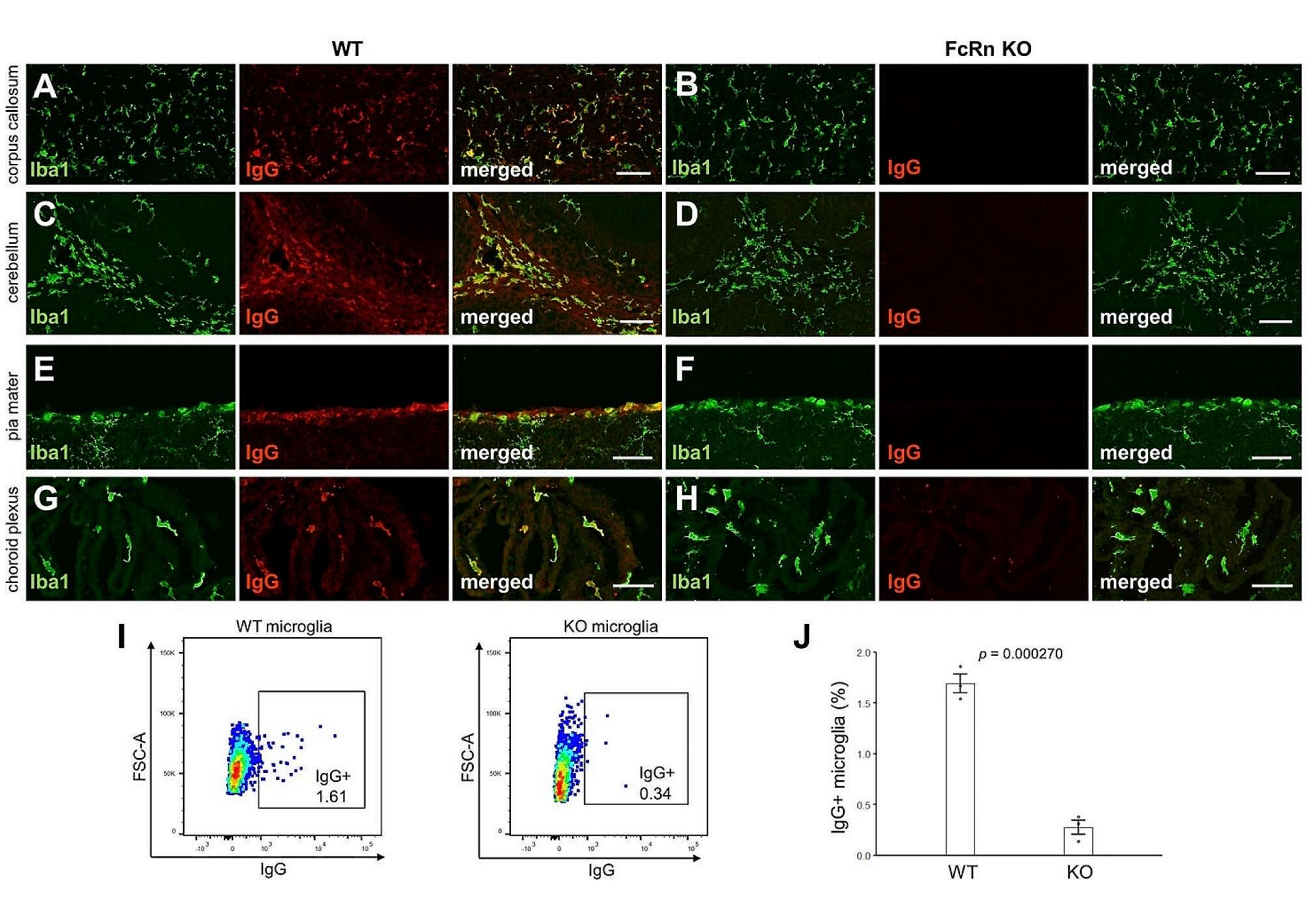
Decreased IgG immunoreactivity in neonatal Fc receptor knockout mice. (**A–D**) Sagittal sections of WT (**A, C**) and FcRn KO (**B, D**) corpus callosum (**A, B**) and cerebellar white matter (**C, D**) at P8 were immunolabeled with an anti-Iba1 (green) and anti-mouse IgG (red) antibodies. Scale bars, 50 μm. (**E–H**) Sagittal sections of WT (**E, G**) and FcRn KO (**F, H**) pia mater (**E, F**) and choroid plexus (**G, H**) at P8 were immunolabeled with an anti-Iba1 antibody (green) and anti-mouse IgG antibody (red). Scale bars, 50 μm. (**I**) Representative flow cytometry plots of P8 microglia stained with anti-mouse IgG antibodies after fixation and permeabilization. (**J**) Flow cytometry quantification of the percentage of P8 microglia stained with anti-mouse IgG after fixation and permeabilization (*n* = 3 for each genotype). The *p*-values from Student’s *t*-test are indicated

IFN-I plays a protective role in neuronal homeostasis, and a lack of IFN-I signaling causes neurodegeneration [44, 45]. To investigate the effects of maternal IgG on infant brain development, we utilized genetically engineered FcRn KO mice. Maternal IgG is transferred from maternal serum into the offspring by FcRn present on placenta and infant intestine [35]. FcRn is primarily expressed in endothelial cells and is also expressed in the respiratory tract upper airway and genitourinary system, and it is mainly involved in the turnover of IgG [35, 46]. Further, FcRn-related brain abnormalities are not found in FcRn KO mice [35, 46]. We compared neonatal brains in offsprings of wild-type (WT; offsprings that received maternal IgG) and FcRn KO mice (offsprings that did not receive maternal IgG). IgG immunoreactivity was observed in the microglia of the corpus callosum of WT mouse brains born from FcRn heterozygote mothers (Fig. 5A) and cerebellar white matter (Fig. 5C). Similarly, the BAMs in the pia mater (Fig. 5E) and choroid plexus (Fig. 5G) also showed IgG immunoreactivity. In the FcRn KO mouse brain, IgG immunoreactivity in these microglia (Fig. 5B and D) and BAMs (Fig. 5F and H) were not observed. The percentage of FcRn KO microglia that showed IgG immunoreactivity was lower than that of WT microglia (Fig. 5I and J).

**Fig. 6:**
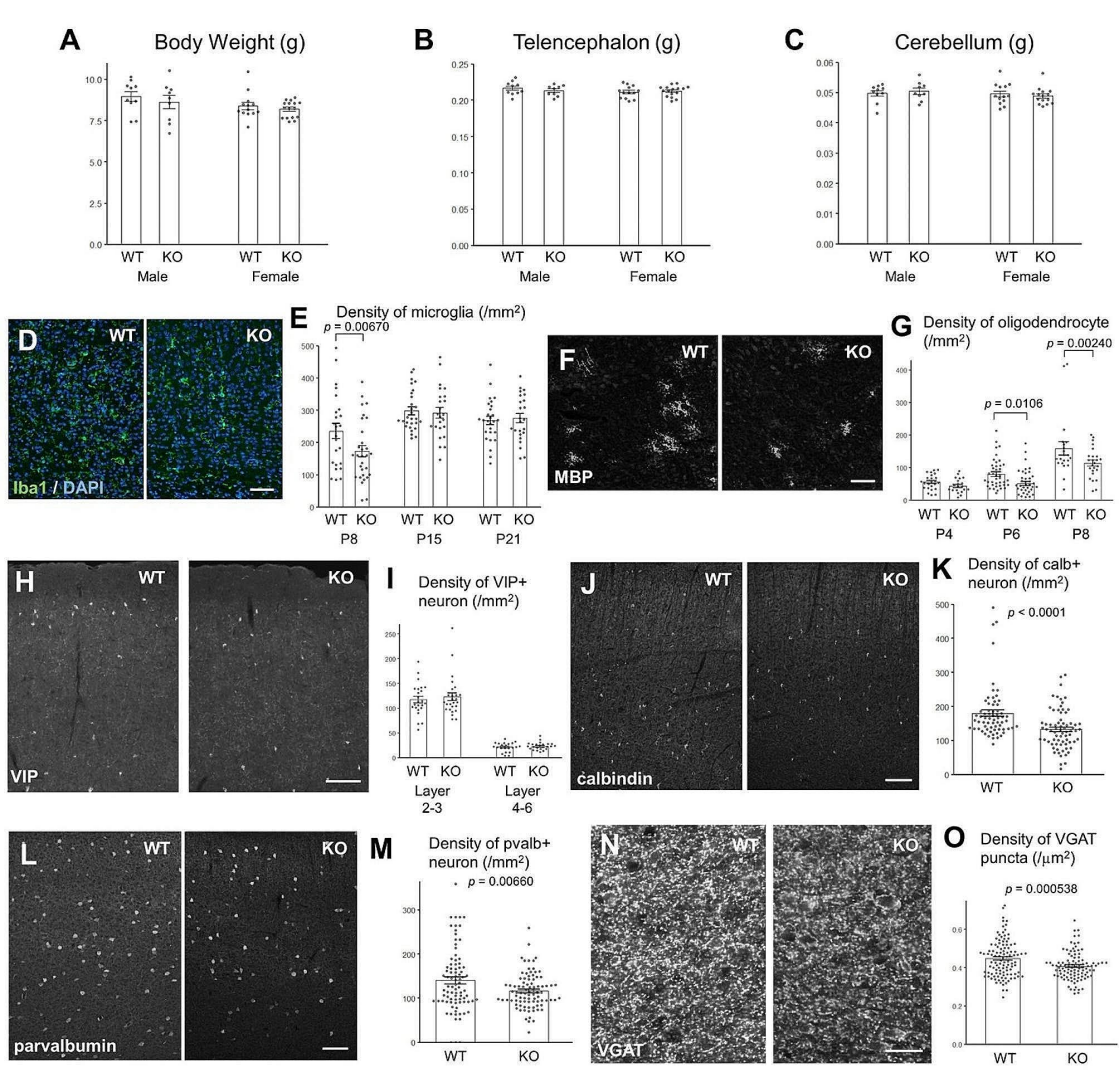
Decreased density of microglia, oligodendrocytes, and neurons in neonatal Fc receptor knockout mice. Body weight (**A**) and wet weight of the telencephalon (**B**) and cerebellum (**C**) at P21 (*n* = 10 WT males; *n* = 10 KO males; *n* = 13 WT females; *n* = 15 KO females). (**D**) Sagittal sections of WT and FcRn KO neocortices at P8, immunolabeled with an anti-Iba1 antibody (green) and 4′,6-diamidino-2-phenylindole (DAPI, blue). Scale bar, 50 μm. (**E**) Density of cells positive for both Iba1 and DAPI in the neocortex [6–8 images were taken per animal; P8 WT (*n* = 24); P8 KO (*n* = 30); P15 WT (*n* = 28); P15 KO (*n* = 24); P21 WT (*n* = 27); P21 KO (*n* = 24)]. A two-way analysis of variance revealed a significant effect of genotype (*p* = 0.0363) and stage (*p* < 0.0001). The *p*-values from Fisher’s PLSD post hoc tests are indicated. (**F**) Sagittal sections of WT and FcRn KO corpus callosum at P6 immunolabeled with an anti-myelin basic protein (MBP) antibody. Scale bar, 50 μm. (**G**) Density of cells positive for MBP (P4 and P6) or myelin-associated glycoprotein (MAG) (P8) in the corpus callosum [Five to eleven images were taken per animal; P4 WT (*n* = 24); P4 KO (*n* = 21); P6 WT (*n* = 41); P6 KO (*n* = 41); P8 WT (*n* = 21); P8 KO (*n* = 25)]. A two-way analysis of variance revealed a significant effect of genotype (*p* = 0.00108) and stage (*p* < 0.0001). The *p*-values from Fisher’s PLSD post hoc tests are indicated. (**H**) Sagittal sections of WT and FcRn KO retrosplenial cortices (RSC) at P21 immunolabeled with an anti-vasoactive intestinal peptide (VIP) antibody. Scale bar, 100 μm. (**I**) Densities of cells positive for VIP in layers 2/3 and layers 4–6 of the RSC [5–7 images were taken per animal; WT layers 2/3 (*n* = 24); KO layers 2/3 (*n* = 26); WT layers 4–6 (*n* = 22); KO layers 4–6 (*n* = 25)]. (**J**) Sagittal sections of WT and FcRn KO RSC at P21 immunolabeled with an anti-calbindin antibody. Scale bar, 100 μm. (**K**) Density of calbindin-positive cells in layer 5 of the RSC [15–19 images were taken per animal; WT (*n* = 66), KO (*n* = 72)]. The *p*-values from Student’s *t*-test are indicated. (**L**) Sagittal sections of WT and FcRn KO RSC at P21, immunolabeled with an anti-parvalbumin (pvalb) antibody. Scale bar, 100 μm. (**M**) Density of cells positive for pvalb in layers 2–6 of the RSC [20–24 images were taken per animal; WT (*n* = 85); KO (*n* = 88)]. The *p*-values from Student’s *t*-test are indicated. (**N**) Sagittal sections of WT and FcRn KO RSC at P21 immunolabeled with an anti-VGAT antibody. Scale bar, 20 μm. (**O**) Density of puncta positive for VGAT in layers 5–6 of the RSC [20–33 images were taken per animal; WT (*n* = 110); KO (*n* = 100)]. The *p*-values from Student’s *t*-test are indicated

No significant changes in the appearance of FcRn KO mice were observed, and no differences in body weight (Fig. 6A) or wet weight of the telencephalon (Fig. 6B) and cerebellum (Fig. 6C) were observed. However, the density of the microglia in the FcRn KO neocortex decreased only at P8 compared with that in the WT neocortex (Fig. 6D and E). The density of oligodendrocytes in the FcRn KO corpus callosum was significantly decreased at P6 and P8 compared with that in the WT corpus callosum (Fig. 6F and G).

Microglia in the corpus callosum showed strong IgG binding activity (Fig. 1A). Because retrosplenial cortex (RSC) is the caudal subdivision of the strip of cortex around the corpus callosum, we immunohistochemically analyzed neurons in the RSC. Rodent studies consistently indicate that the RSC plays a crucial role in learning and navigation, thought to be orchestrated in collaboration with various thalamic and hippocampal systems [47, 48]. Moreover, ketamine treatment increases neural activity in the RSC and decreases social behaviors in mice [49]. We found that the density of vasoactive intestinal peptide (VIP)-positive interneurons did not differ among the cortical areas examined at P21 (Fig. 6H and I). In contrast, at P21, the density of calbindin- (Fig. 6J and K) and parvalbumin- positive interneurons (Fig. 6L and M) in RSC region of FcRn KO mouse brain was significantly decreased compared with that in RSC region of WT mouse brain. The density of the vesicular γ-Aminobutyric acid (GABA) transporter (VGAT), a marker of inhibitory synapses, also decreased in RSC region of FcRn KO mouse brain, at P21 (Fig. 6N and O). These changes were persistent at postnatal age of 4 months. In 4 month old mice, we found similar decreases in parvalbumin- and calbindin- positive interneurons, and VGAT puncta in RSC region of FcRn KO mice brain (Fig. S7).

**Fig. 7:**
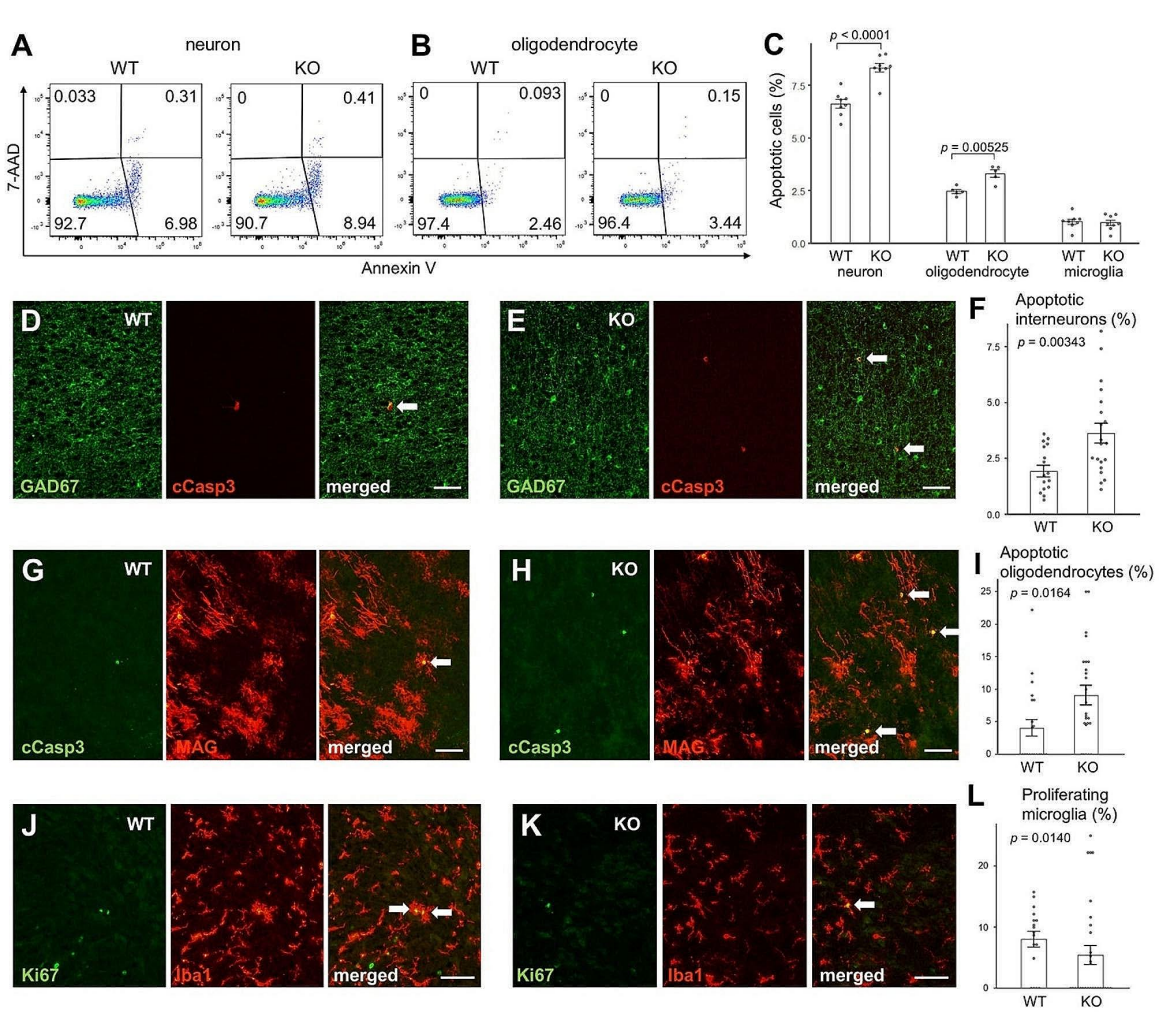
Increased apoptotic neurons and oligodendrocytes and decreased proliferative microglia in neonatal Fc receptor knockout brain. (**A, B**) Apoptotic neurons (CD200+/CD45−/CD31− cells) (**A**) and O4-positive oligodendrocytes (**B**) at P12. (**C**) The percentage of early apoptotic (annexin+/7-AAD−) neurons, oligodendrocytes, and microglia at P12. Cell debris was eliminated by initial gating, and 7-AAD-positive cells were excluded because neurons and oligodendrocytes are easily damaged during the process of isolation from the brain. WT neuron (*n* = 8); KO neuron (*n* = 8); WT oligodendrocyte (*n* = 5); KO oligodendrocyte (*n* = 5); WT microglia (*n* = 8); KO microglia (*n* = 8). A two-way analysis of variance revealed a significant effect of genotype (*p* < 0.0001) and stage (*p* < 0.0001). The *p*-values from Fisher’s PLSD post hoc tests are indicated. (**D, E**) Sagittal sections of WT (**D**) and FcRn KO (**E**) RSC at P8 immunolabeled with anti-GAD67 (green) and anti-cleaved caspase3 (red) antibodies. White arrows point to the double-positive cells. Scale bar, 50 μm. (**F**) The percentage of apoptotic cells in GAD67-positive interneurons in the P8 RSC [4–5 images were taken per animal; WT (*n* = 17); KO (*n* = 20)]. The *p*-values from Student’s *t*-test are indicated. (**G, H**) Sagittal sections of WT (**G**) and FcRn KO (**H**) corpus callosum at P8 immunolabeled with anti-cleaved caspase3 (green) and anti-MAG (red) antibodies. White arrows point to the double-positive cells. Scale bar, 50 μm. (**I**) Percentage of apoptotic cells in MAG-positive oligodendrocytes in the P8 corpus callosum [5–7 images were taken per animal; WT (*n* = 21); KO (*n* = 25)]. The *p*-values from Student’s *t*-test are indicated. (**J, K**) Sagittal sections of the WT (**J**) and FcRn KO (**K**) corpus callosum at P8 immunolabeled with anti-Ki67 (green) and anti-Iba1 (red) antibodies. White arrows point to the double-positive cells. Scale bar, 50 μm. (**L**) The percentage of Ki67-positive proliferating cells in microglia in the P8 corpus callosum [5–7 images were taken per animal; WT (*n* = 23), KO (*n* = 26)]. The *p*-values from Student’s *t*-test are indicated

CD200 (OX2) is mainly expressed in central nervous system (CNS) neurons [50]. However, because it is also expressed in lymphocytes and endothelial cells [50], we investigated neural apoptosis using flow cytometry in the CD200+/CD45−/CD31− population. Because neurons and oligodendrocytes are intricately intertwined, cells are easily destroyed during the process of isolation for flow cytometric analysis. Therefore, only early apoptotic cells (7-AAD-negative and annexin V-positive cells) were quantified after gating out cell debris. The results showed that the density of apoptotic neurons and oligodendrocytes was higher in the FcRn KO infant brain than in the WT brain (Fig. 7A and B, and 7C). In contrast, no difference in the percentage of apoptotic microglia was observed between the control and FcRn KO brains (Fig. 7C). The immunohistochemistry results showed that the percentage of apoptotic GAD67-positive interneurons in the FcRn KO neocortex was higher than that in the WT neocortex (Fig. 7D and E, and 7F). Similarly, the percentage of apoptotic oligodendrocytes in the FcRn KO corpus callosum was higher than that in the WT corpus callosum (Fig. 7G and H, and 7I). In contrast, the density of Ki67-positive proliferating microglia in the WT corpus callosum was higher than that in the FcRn KO corpus callosum (Fig. 7J and K, and 7L).

### Behavioral phenotypes of FcRn KO offspring

To examine the effects of maternal IgG on brain development in offsprings, a comprehensive behavioral analysis of FcRn KO mice was performed. No significant differences in body temperature, grip strength, latency to fall off the wire grid, motor function assessed using the rotarod test, or pain sensitivity examined using the hot plate test were observed between WT and FcRn KO mice (Fig. S8).

**Fig. 8:**
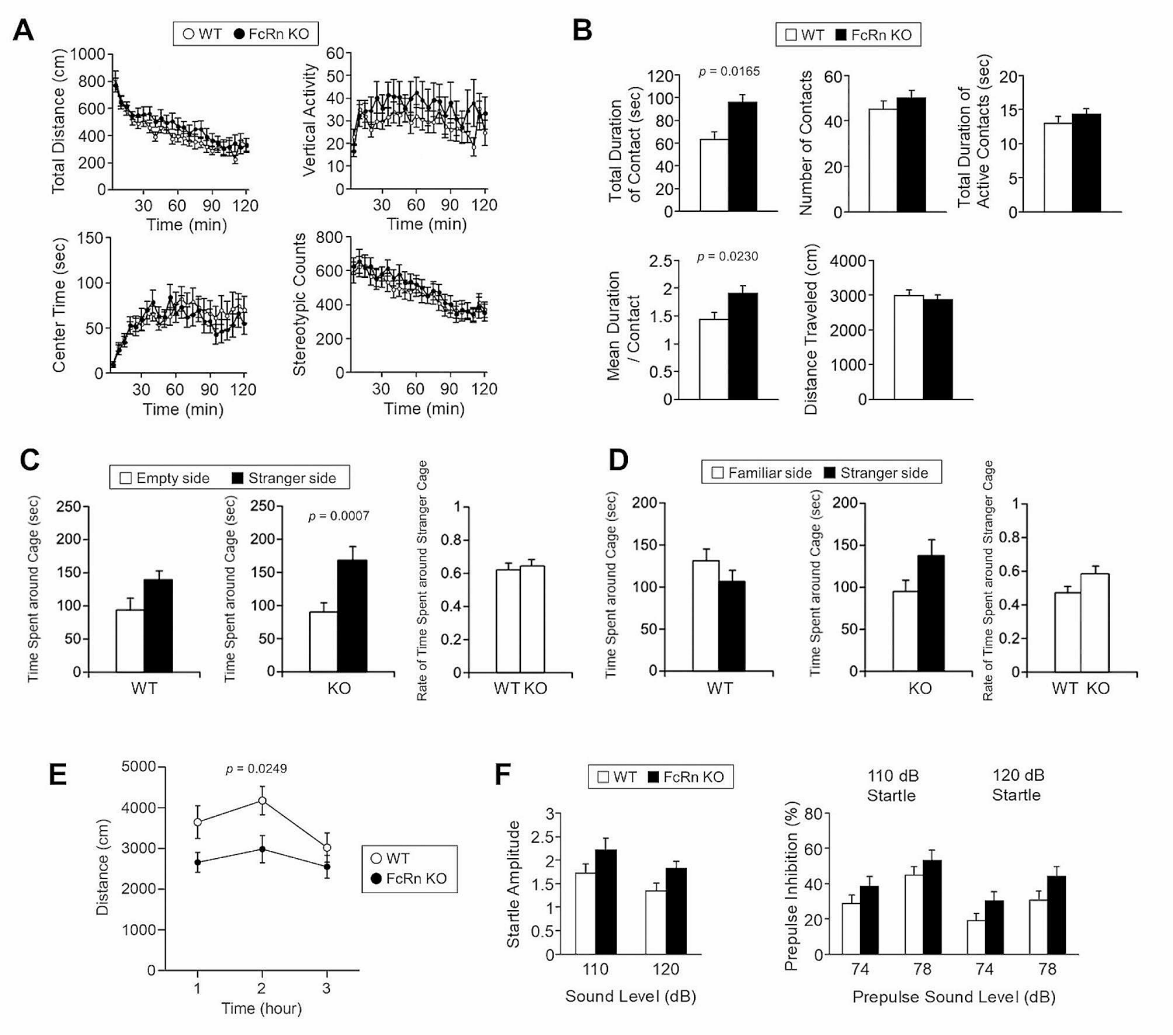
Abnormal behavioral phenotypes of neonatal Fc receptor knockout mice. (**A**) Total distance traveled, vertical activity, time spent in the center area, and stereotypical behavior counts for WT (open circles, *n* = 20) and FcRn KO mice (closed circles, *n* = 20) are represented.(**B**) Social interaction test in novel environments, total duration of contacts, number of contacts, total duration of active contacts, mean duration per contact, and total distance traveled by WT (open columns, *n* = 10 pairs) and FcRn KO mice (closed columns, *n* = 10 pairs) are presented. The *p*-values indicate the genotype effects in one-way ANOVA. (**C, D**) Crawley’s sociability and social novelty preference test. Time spent around the cage and the rate of time spent around the stranger cage in the sociability (**C**) and social novelty preference (**D**) tests [WT (*n* = 20); KO (*n* = 20)]. The *p*-values indicate the genotype effects in one-way ANOVA. (**E**) Home-cage locomotor activity of WT (open circles, *n* = 17) and FcRn KO mice (closed circles, *n* = 19). The *p*-values indicate genotype effects in the two-way repeated-measures ANOVA. (**F**) Startle amplitude and percent of pre-pulse inhibition in WT (open columns, *n* = 20) and FcRn KO mice (closed columns, *n* = 20)

The locomotor activity of FcRn KO mice was evaluated using the open field test. No significant differences in the total distance traveled, vertical activity, time spent in the center area, or stereotypic behaviors were observed between the genotypes (Fig. 8A). In the novel environment open field social interaction test, FcRn KO mice exhibited a significant increase in the total duration of contact (Fig. 8B). The mean duration per contact was also significantly increased in FcRn KO mice (Fig. 8B). The mice were also subjected to Crawley’s sociability and social novelty preference test, which comprises a sociability test and a social novelty preference test. In the sociability test, social behavior is assessed based on the time spent around a wire cage with an unfamiliar mouse (stranger side) *versus* the time spent around an empty cage (empty side). Both WT and FcRn KO mice spent more time around the stranger-side cage than around the empty-side cage, and no difference in the time spent around the stranger side was observed between the genotypes (Fig. 8C). In the social novelty preference test, FcRn KO mice stayed longer around the stranger side of the cage than WT mice; however, the difference was statistically insignificant (*p* = 0.0721) (Fig. 8D). Figure 8E shows that FcRn KO mice exhibited less locomotor activity than WT mice during the night in the home cage, which was different from that in the open field (Fig. 8A).

Pre-pulse startle inhibition is an operational measure of sensorimotor gating; it is often impaired in patients with schizophrenia [51]. Acoustic startle responses were similar in WT and FcRn KO mice (Fig. 8F). Although the percentage of prepulse inhibition was higher in FcRn KO mice than in WT mice at 120 dB, the difference did not reach statistical significance (*p* = 0.0812) (Fig. 8F).

## Discussion

Here, we showed that maternal IgG immunoreactivity is observed in the offspring brain microglial cells, peaking at P8 (Fig. 1). IgG stimulation of primary cultured microglia activated the IFN-I feedback loop by Syk (Figs. 2 and 3). IgG2a and IgG2b were found to exhibit these activities in the Fc region alone (Fig. 4). Analysis of FcRn KO mice showed that the density of microglia was decreased only in the infant brain (Fig. 6D), whereas the number of calbindin- and parvalbumin-positive interneurons decreased in later developmental stages (Fig. 6 and S7). FcRn KO mice also exhibited abnormalities in social behavior and locomotor activity in their home cages (Fig. 8). This study suggests that maternal IgG affects brain development in offspring.

Parvalbumin cells have been suggested to be associated with sociability [52, 53]. Mice with reduced (parvalbumin+/−) or absent (parvalbumin−/−) parvalbumin expression levels show behavioral deficits related to human autism spectrum disorder core symptoms [54]. Moreover, dysfunction in parvalbumin cells has been observed in various mouse models of autism [55–59]. Interestingly, the number of parvalbumin-expressing interneurons is decreased in the prefrontal cortex of patients with autism [60]. Moreover, stimulating parvalbumin cells has been shown to be sufficient to improve social behavior [56, 61]. FcRn KO mice showed a decreased density of parvalbumin-positive interneurons (Fig. 6L and M, S7E, and S7F) and increased duration of contact during the social interaction test (Fig. 8B). However, it is difficult to conclude that the absence of IgG from the mother improves sociability because some cases in which autism mouse models exhibit behavior similar to that of FcRn KO mice have been reported. Shank2 KO mice show increased social interactions following isolation [62]. Mice with heterozygous Chd8 mutations exhibit prolonged social interaction duration in tests [63]. Mice lacking intergenic Dlx enhancers also show increased social interaction [64]. These reports suggest that the increased duration of interaction in the social interaction test is a social behavior impairment. On the other hand, an increased duration of contact is generally considered an increase in sociability. Is it better for the offspring’s brain if there is no IgG transfer from the mother to the infant? Epidemiological studies have shown an association between maternal infection and a child’s subsequent risk of autism and schizophrenia [65, 66]. Analyses of maternal immune activation (MIA) mouse models have shown that maternal interleukin (IL)-17a promotes autistic behavior in pups [67]. In MIA mouse models, it is probable that increased maternal IgG is transferred to pups. The effect of abnormally increased IgG levels in the maternal blood on the social behavior of offspring should also be elucidated in the future.

The binding of IgG to neurons [68], oligodendrocytes [69], and microglia [43] has been previously reported. Nakahara et al. reported IgG immunoreactivity in oligodendrocytes [69] in the infant mouse brain. In contrast, Hazama et al. demonstrated IgG immunoreactivity in microglia and neurons in the adult mouse brain [43]. In this report, no IgG immunoreactivity was observed in cell types other than the microglia (Fig. 1). Hazama et al. also reported species differences in IgG immunoreactivity in the brains of various rodents and no IgG immunoreactivity in CBA/N and CBA/J mice [43]. In contrast, we observed IgG immunoreactivity in the brains of infant CBA/N mice (Fig. S6A). Moreover, upon stimulation with IgG, Stat1 was phosphorylated in primary cultured microglia prepared from CBA/N mice (Fig. S6B) and the human microglial HMC3 cell line (Fig. S6C). When observing IgG immunoreactivity using immunohistochemistry, “diffusion artifacts” can cause false-positive results [70, 71]. The diffusion of components is usually caused by the translocation of serum proteins, such as immunoglobulins, from the extracellular matrix into the cytoplasm of some cells because ischemia during the preparation of tissue specimens is assumed to quickly change cell membrane permeability in animal organs. Therefore, we examined IgG immunoreactivity using flow cytometry and Stat1 phosphorylation by IgG stimulation and confirmed that IgG immunoreactivity in the microglia was not an artifact.

We performed a detailed analysis of the effects of IgG in breast milk on the neonatal brain using immunohistochemistry, flow cytometry and behavioral analyses. No reports have shown the behavioral phenotypes of FcRn KO mice or the immunohistochemical phenotypes of FcRn KO brains. On the other hand, knockout of the γ-chain subunit decreases the number of myelin-associated glycoprotein (MAG)-positive oligodendrocytes in the brain of infant mice [69], which is similar to our results (Fig. 6G). It has also been reported that knockout of the γ-chain subunit reduces hippocampal pyramidal cell death induced by kainic acid [72]. IgG Fc receptors have three classes: FcγRI, FcγRII, and FcγRIII [73]. The γ-chain is a subunit of FcγRI and FcγRIII. γ-chains are also a shared subunit of FcεRI [73]. The differences in the deleted classes should be carefully analyzed in the future to determine what phenotypic differences may result from the differences.

How does maternal IgG reach the brains of pups? In the postnatal mouse brain, the blood–brain barrier (BBB) is considered complete [74]. However, not all CNS vascularities possess a BBB; exceptions include the pineal gland, neurohypophysis, area postrema, subfornical organ, median eminence, and vascular organ of the lamina terminalis [74]. These regions are referred to as circumventricular organs and permit the diffusion of blood-borne molecules [75]. On the other hand, incidental intracranial hemorrhages are relatively common among human infants born by vaginal delivery [76]. Although whether this phenomenon occurs in mice is unclear, it is thought to be one of the candidate pathways through which IgG migrates to the brain. It is also possible that FcRn transports IgG across blood–brain barrier (BBB). However, the research on this is not settled. Intracranially applied antibody is exported to the blood across the BBB by FcRn-mediated transcytosis [77, 78]. In contrast, studies using the systemic application of antibodies suggest that FcRn is irrelevant for brain-to-blood export [79–82]. Further, a substantial antibody deposition in brain was observed upon the systemic administration of a modified human IgG1 FcRn-high-binding variant [83]. Furthermore, this effect was abolished in FcRn KO mice and when the unmodified human IgG1 antibody was used. These findings suggest that when excess IgG is administered to the brain, IgG is exported from the brain in an FcRn-dependent manner, but otherwise, FcRn may be involved in bidirectional transport across the brain-blood barrier in all other conditions. In future, we will determine whether FcRn is involved in the bidirectional transport of IgG across BBB by using FcRn conditional KO mice.

During postnatal development, microglia contribute to the survival of neocortical neurons by releasing IGF-1 as a trophic factor [12]. IGF-1 also directly acts on oligodendrocytes and myelination, as demonstrated in oligodendrocyte lineage-specific KO mice of the IGF-1R [84]. Other factors produced by microglia have also been found to support the health and maturation of oligodendrocyte precursor cells and mature oligodendrocytes in the developing CNS [85]. For example, microglia-derived IGF-2 has been shown to prevent tumor necrosis factor-α-induced apoptosis of oligodendrocytes [86]. However, no difference in the expression of IGF-1 or IGF-2 was observed between the control and IgG-stimulated primary cultured microglia in our study (Fig. 2). IFN-I also plays a protective role in neuronal homeostasis, and a lack of IFN-β signaling increases the expression of multiple genes annotated as neuronal degeneration and increases neuronal apoptosis [44, 45]. IgG stimulation of primary cultured microglia activated the IFN-I feedback loop (Figs. 2 and 3). The effects of microglia on oligodendrocytes and neurons via IFN-I and/or trophic factors should be examined in detail in future studies.

A phagocytic/activated microglial phenotype, referred to as DAM/ARM/MGnD, has been recognized in mouse models of Alzheimer’s disease (AD) featuring amyloid pathology [87–89]. Single-cell RNA-seq (scRNA-seq) has further resolved DAM/ARM/MGnD complexity, identifying four microglial signatures in amyloid pathology, namely, DAM/ARM/MGnD proper (enriched for ApoE, Clec7a, Cst7, and Lpl), MHC class II microglia (enriched for MHC-II molecules), proliferating microglia, and IFN-I-responsive microglia (enriched for Ifit2, Irf7, Oasl2, and Stat1) [89–91]. In this study, we showed that Syk is upstream of the IFN-I feedback loop (Fig. 3H). Deletion of Syk gene in microglia reduces the proportion of IFN-I-responsive microglia, and DAP12 is an upstream candidate for Syk in the AD brain [92]. Syk may also bind to the IgG receptor FcγR because disruption of the BBB may allow IgG to enter the brain in AD [93]. Whether Syk-mediated activation of the IFN-I feedback loop occurs in the AD brain would be interesting to examine.

Here, we showed that changes in the mother-derived IgG levels affect neonatal brain development. Immune-related molecules, including major histocompatibility complex class I (MHC I), CD3zeta, and IgM, contribute to brain development [94–96]. The effects of immune-related molecules on brain development remains to be comprehensively elucidated.

### Electronic supplementary material

Below is the link to the electronic supplementary material.

Supplementary Material 1

## Data Availability

The data of RNA-Seq presented in this manuscript have been deposited in NCBI’s Gene Expression Omnibus and are accessible through the GEO Series accession number GSE248310.

## Abbreviations

AD: Alzheimer’s disease
APC: Allophycocyanin
BAM: Border-associated macrophage
BBB: Blood–brain barrier
DEG: Differentially expressed gene
IFN-I: Type I interferon
IL: Interleukin
ISG: Interferon-stimulated gene
MAG: Myelin-associated glycoprotein
MBP: Myelin basic protein
PE: Phycoerythrin
PerCP: Peridinin chlorophyll protein
PPI: Prepulse inhibition
VGAT: Vesicular γ-Aminobutyric acid (GABA) transporter
VIP: Vasoactive intestinal peptide
