# Supplementary material for: Maternal immunoglobulin G affects brain development of mouse offspring
Source: J Neuroinflammation. 2024 May 2;21:114. doi: 10.1186/s12974-024-03100-z (PMC11064405; doi:10.1186/s12974-024-03100-z)
Supplement: Supplementary file 1 — Supplementary Material 1 [file 12974_2024_3100_MOESM1_ESM.docx]

**SUPPLEMENTARY INFORMATION**

Maternal Immunoglobulin G Affects Brain Development of Mouse Offsprings

Mizuki Sadakata^1,*^, Kazuki Fujii^2,3,4^, Ryosuke Kaneko^5^, Emi Hosoya^1^, Hisako Sugimoto^1^, Reika Kawabata-Iwakawa^6^, Tetsuhiro Kasamatsu^7^, Shoko Hongo^4^, Yumie Koshidaka^4^, Akinori Takase^8^, Takatoshi Iijima^9^, Keizo Takao^2,3,4^, Tetsushi Sadakata^1,**^

^1^Education and Research Support Center, Gunma University Graduate School of Medicine, Maebashi, Gunma 371-8511, Japan

^2^Department of Behavioral Physiology, Faculty of Medicine, University of Toyama, Sugitani, Toyama, 930-0194, Japan

^3^Research Center for Idling Brain Science, University of Toyama, Sugitani, Toyama, 930-0194, Japan

^4^Life Science Research Center, University of Toyama, Sugitani, Toyama, 930-0194, Japan

^5^KOKORO-Biology Group, Graduate School of Frontier Biosciences, Osaka University, Suita, Osaka 565-0871, Japan

^6^Division of Integrated Oncology Research, Gunma University Initiative for Advanced Research (GIAR), Gunma University, Maebashi, Gunma 371-8511, Japan

^7^Department of Laboratory Sciences, Gunma University Graduate School of Health Sciences, Maebashi, Gunma 371-8514, Japan

^8^The Support Center for Medical Research and Education, Tokai University, Isehara, Kanagawa 259-1193, Japan

^9^Institute of Innovative Science and Technology, Tokai University, Isehara, Kanagawa 259-1193, Japan.

^*^Corresponding author. Tel: +81 27 220 8299; E-mail: m2200038@gunma-u.ac.jp

^**^Corresponding author. Tel: +81 27 220 8299; E-mail: sadakata-1024@gunma-u.ac.jp

**
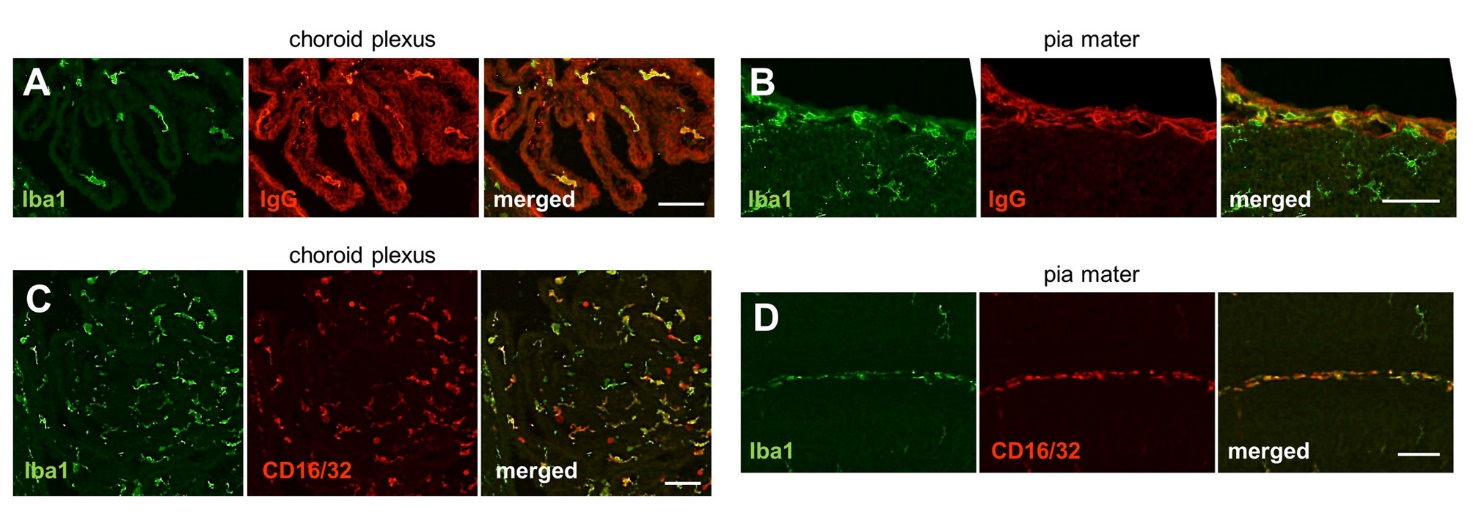
**

**Supplementary Fig. 1. IgG and CD16/32 immunoreactivity in the border-associated macrophages**

**(A, B)** Sagittal sections of choroid plexus (A) and pia mater (B) at P8 were immunolabeled with anti-Iba1 (green) and anti-mouse IgG (red) antibodies. Scale bars, 50 µm. **(C, D)** Sagittal sections of choroid plexus (C) and pia mater (D) at P8 were immunolabeled with anti-Iba1 (green) and anti-CD16/32 IgG (red) antibodies. Scale bars, 50 µm.

**
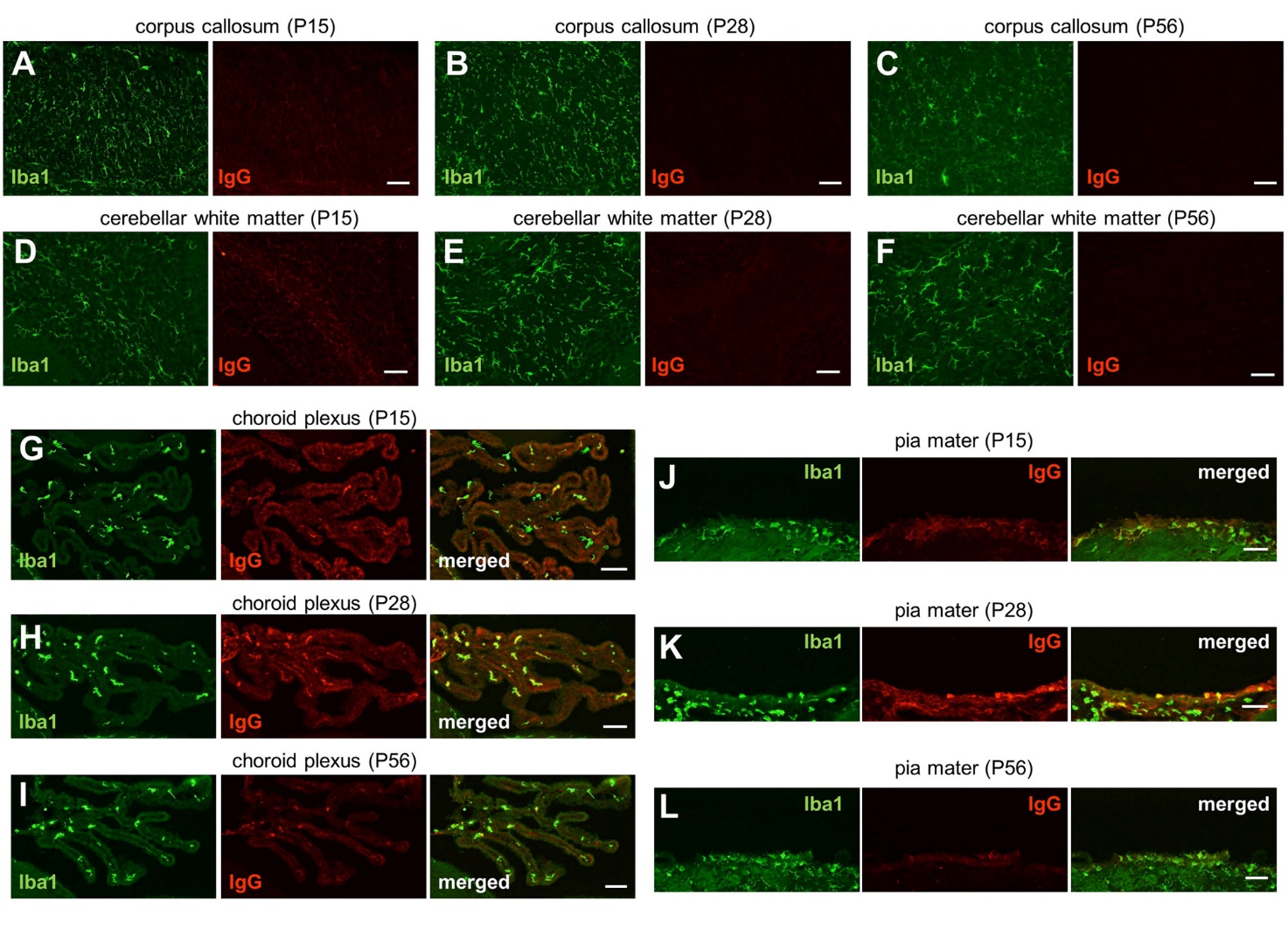
**

**Supplementary Fig. 2. IgG immunoreactivity in microglia and** **border-associated macrophages at P15, P28, and P56**

**(A–F)** Sagittal sections of the corpus callosum (A, B, C) and cerebellar white matter (D, E, F) were immunolabeled with anti-Iba1 (green) and anti-mouse IgG (red) antibodies at P15 (A, D), P28 (B, E), and P56 (C, F). Scale bars, 50 µm. **(G–L)** Sagittal sections of the choroid plexus (G, H, I) and pia mater (J, K, L) were immunolabeled with anti-Iba1 (green) and anti-mouse IgG (red) antibodies at P15 (G, J), P28 (H, K), and P56 (I, L). Scale bars, 50 µm.


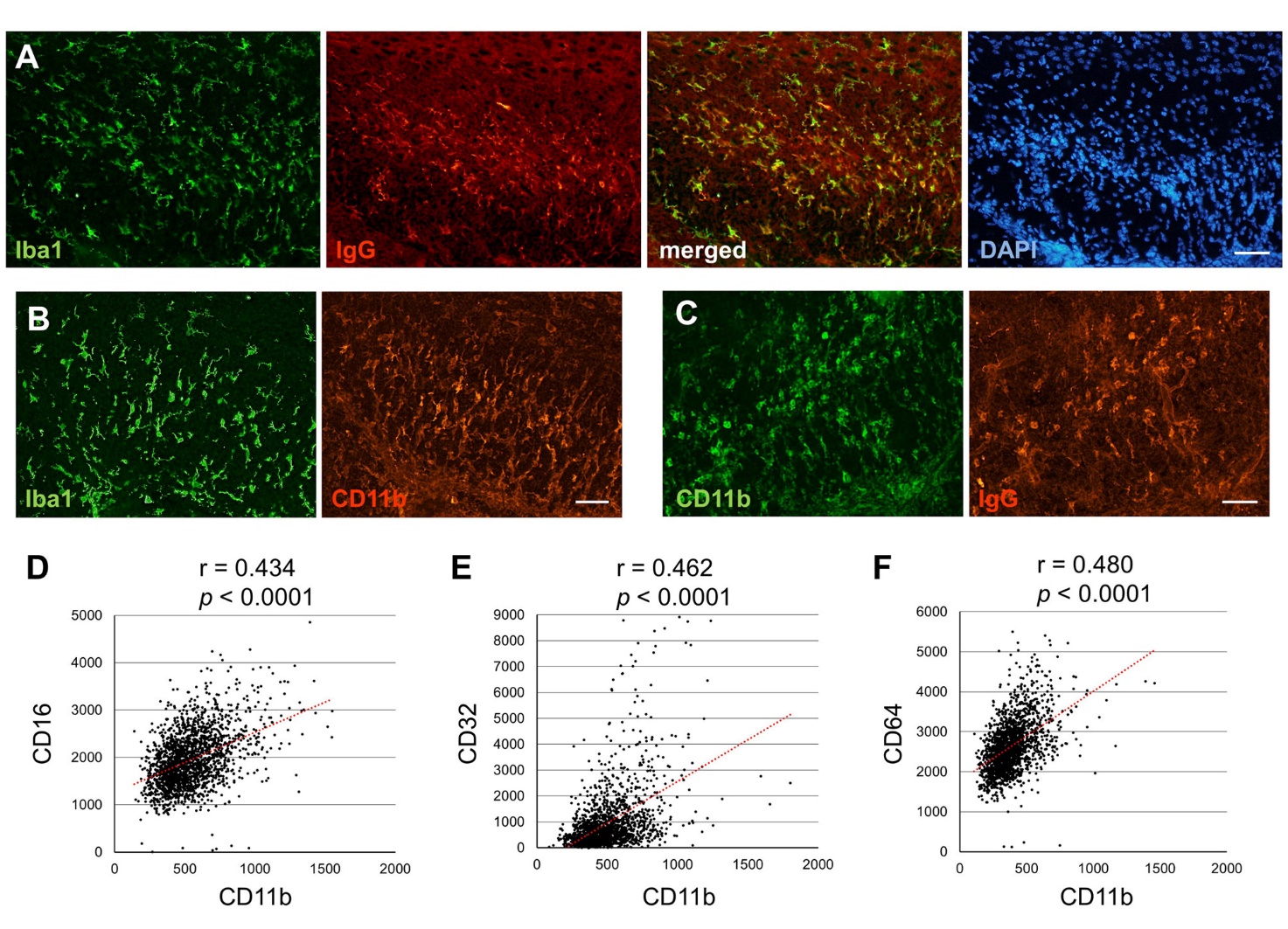


**Supplementary Fig. 3. Heterogeneity of CD11b expression in the microglia**

**(A)** Sagittal sections of the corpus callosum were immunolabeled with anti-Iba1 (green) and anti-mouse IgG (red) antibodies and DAPI (blue) at P8. Scale bars, 50 µm. **(B)** Sagittal sections of the corpus callosum were immunolabeled with anti-Iba1 (green) and anti-CD11b (red) antibodies at P8. Scale bars, 50 µm. **(C)** Sagittal sections of the corpus callosum were immunolabeled with anti-CD11b (green) and anti-mouse IgG (red) antibodies at P8. Scale bars, 50 µm. **(D–F)** Correlation between per microglia fluorescence intensity of CD11b and CD16 (D), CD32 (E), and CD64 (F) in the P8 mouse brain [CD16 (*n* = 1912); CD32 (*n* = 1779); CD64 (*n* = 1826)]. r = Spearman correlation coefficient.


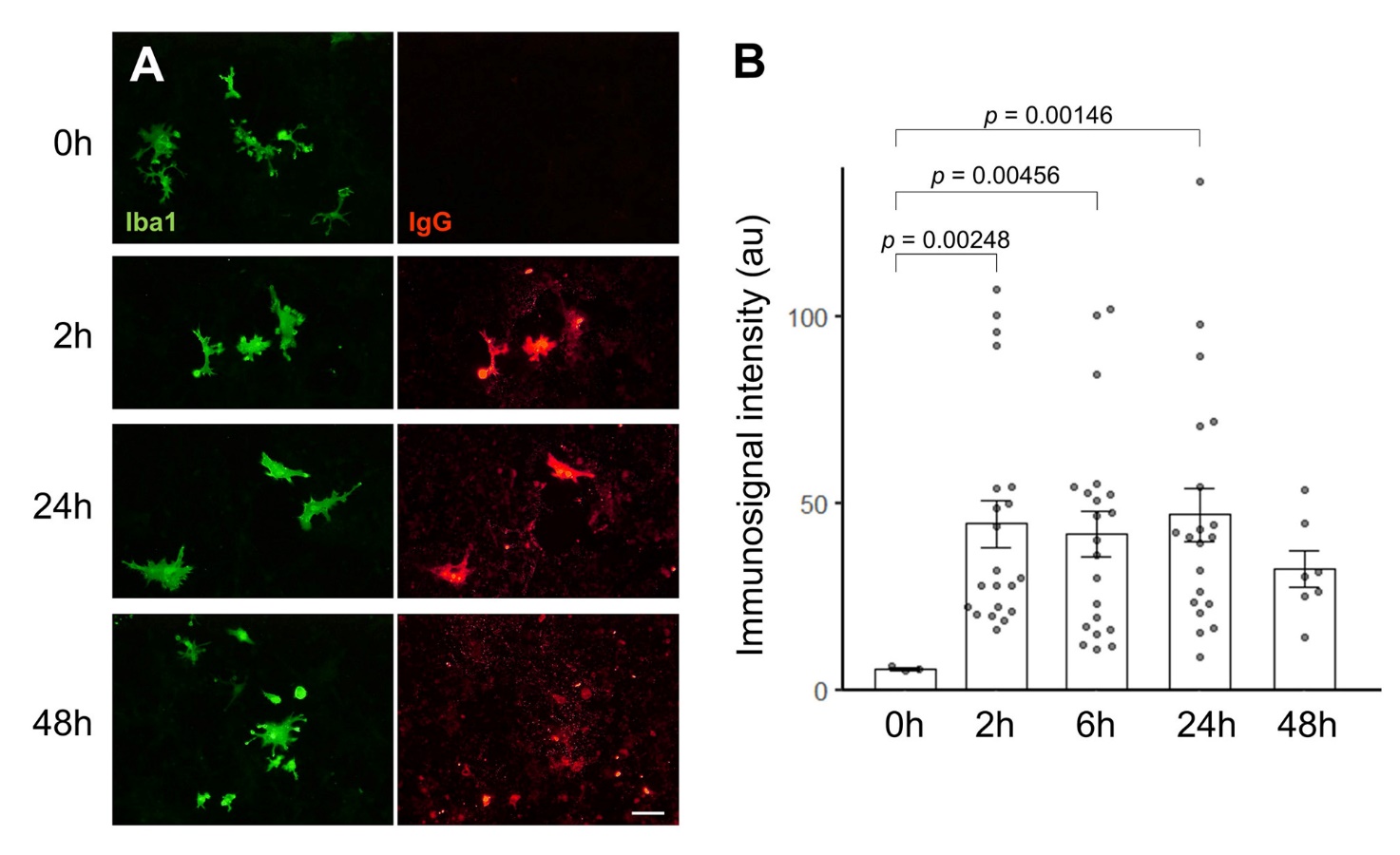


**Supplementary Fig. 4. IgG immunoreactivity of microglia after treatment of IgG**

**(A)** Primary cultured microglia treated with and without IgG were immunolabeled with anti-Iba1 (green) and anti-mouse IgG (red) antibodies. The first panel (0h) represents the immunoreactivity before the addition of IgG, and the lower three panels represent the immunoreactivity 2 h, 24 h, and 48 h after the addition of 10 µg/ml IgG to the culture media. Scale bars, 50 µm. **(B)** IgG immunoreactivity was measured as fluorescence intensity in arbitrary units (au) before adding IgG and 2 h, 6 h, 24 h, and 48 h after the addition of 10 µg/ml IgG to the culture media [0 h (*n* = 3); 2 h (*n* = 21); 6 h (*n* = 21); 24 h (*n* = 20); 48 h (*n* = 7)]. One-way analysis of variance showed a significant difference (*p* = 0.0148). The *p*-values from Fisher’s PLSD post hoc tests are indicated.


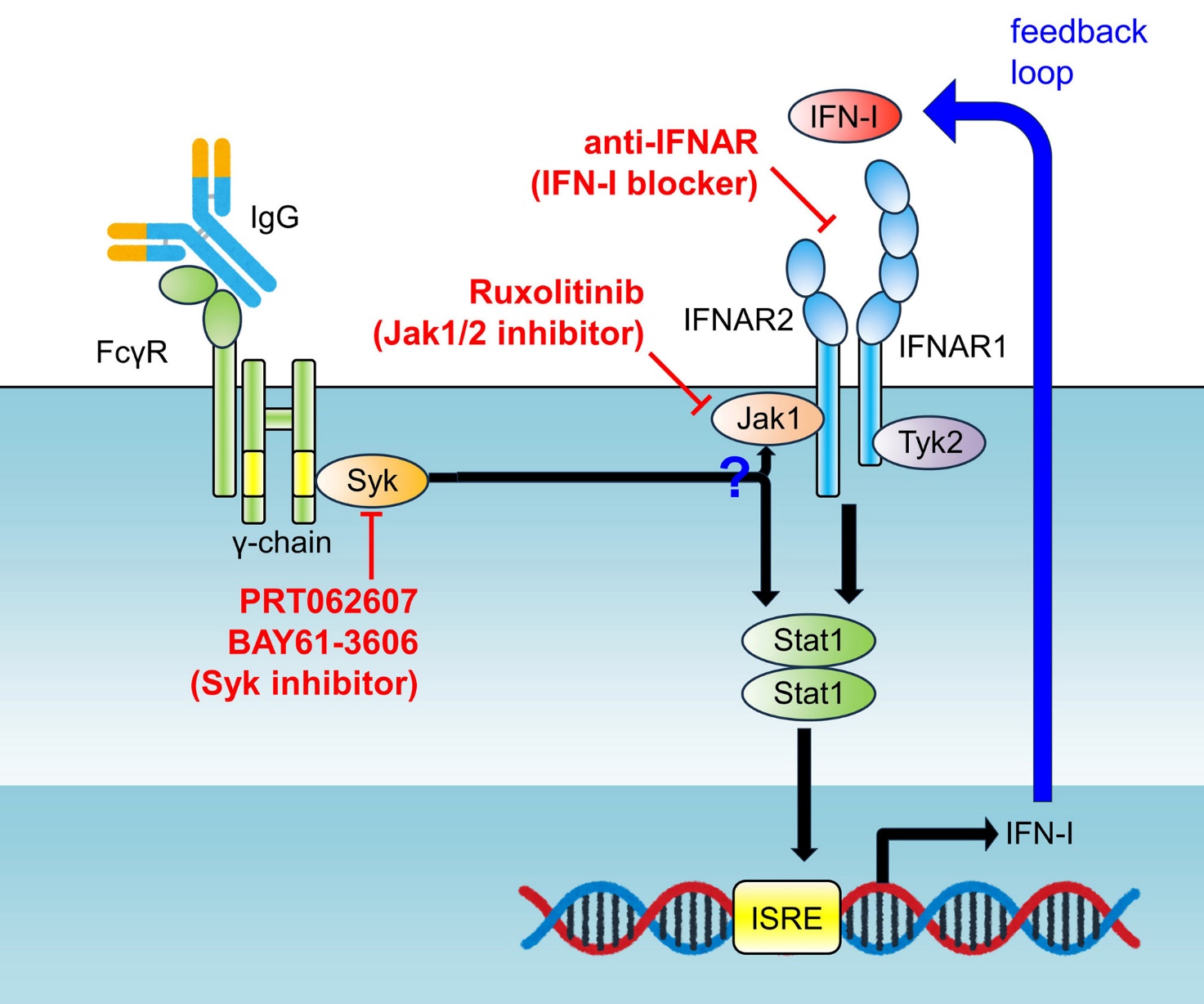


**Supplementary Fig. 5. Model for the activated IFN-I feedback loop mediated by Syk**

Inhibitors associated with the signaling pathway are indicated in red.


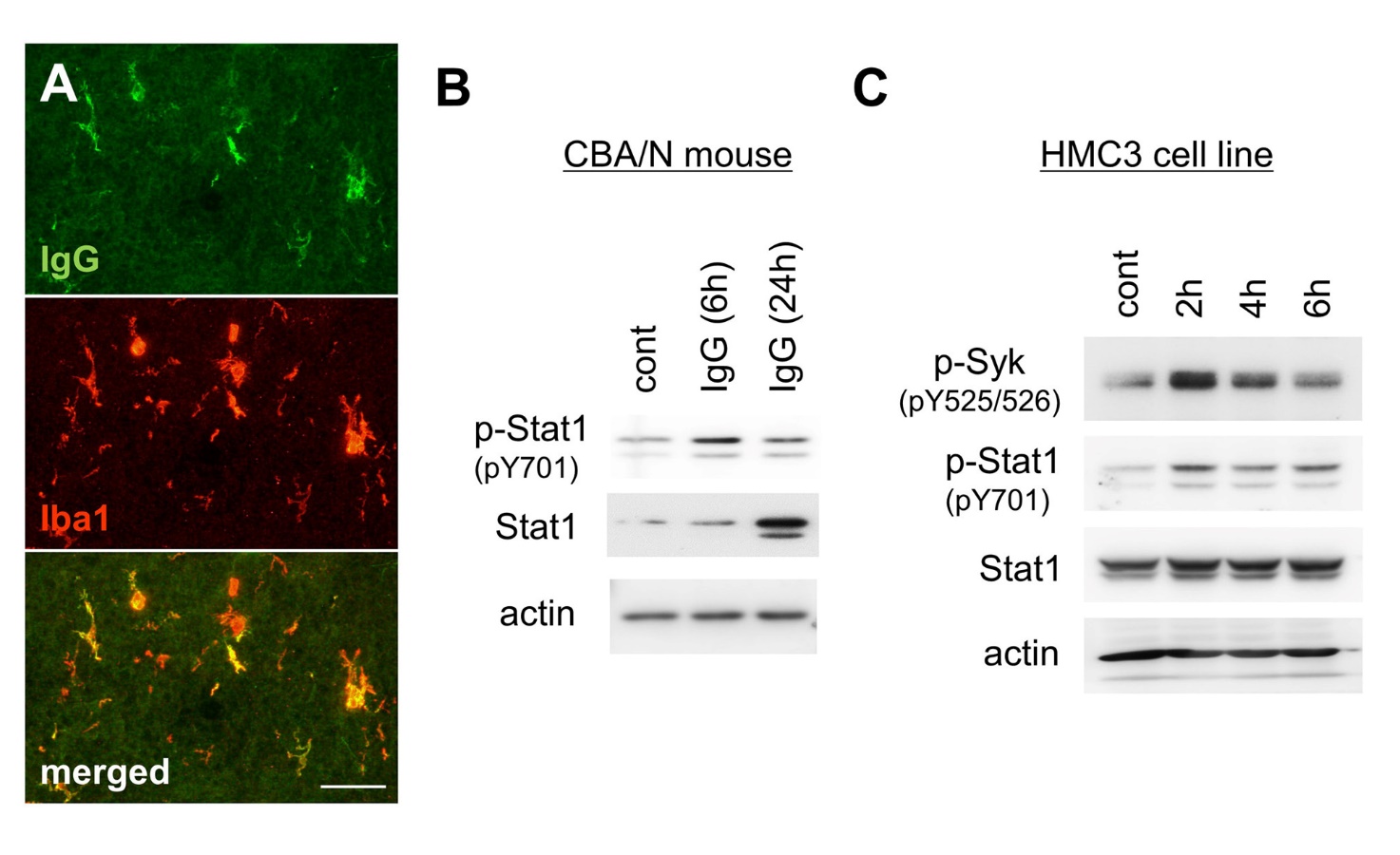


**Supplementary Fig. 6. Response to IgG stimulation in microglia of CBA/N mice and human microglial cell line**

**(A)** The corpus callosum of CBA/N mice at P8 was immunolabeled with anti-mouse IgG (green) and anti-Iba1 (red) antibodies. Scale bars, 40 µm. **(B)** Immunoblot analysis of primary cultured microglia prepared from CBA/N mice. Whole-cell extracts of microglia stimulated with 10 µg/ml mouse IgG were analyzed. **(C)** Immunoblot analysis of the human microglial cell line HMC3. Whole-cell extracts of HMC3 cells stimulated with 100 µg/ml human IgG were analyzed.

**
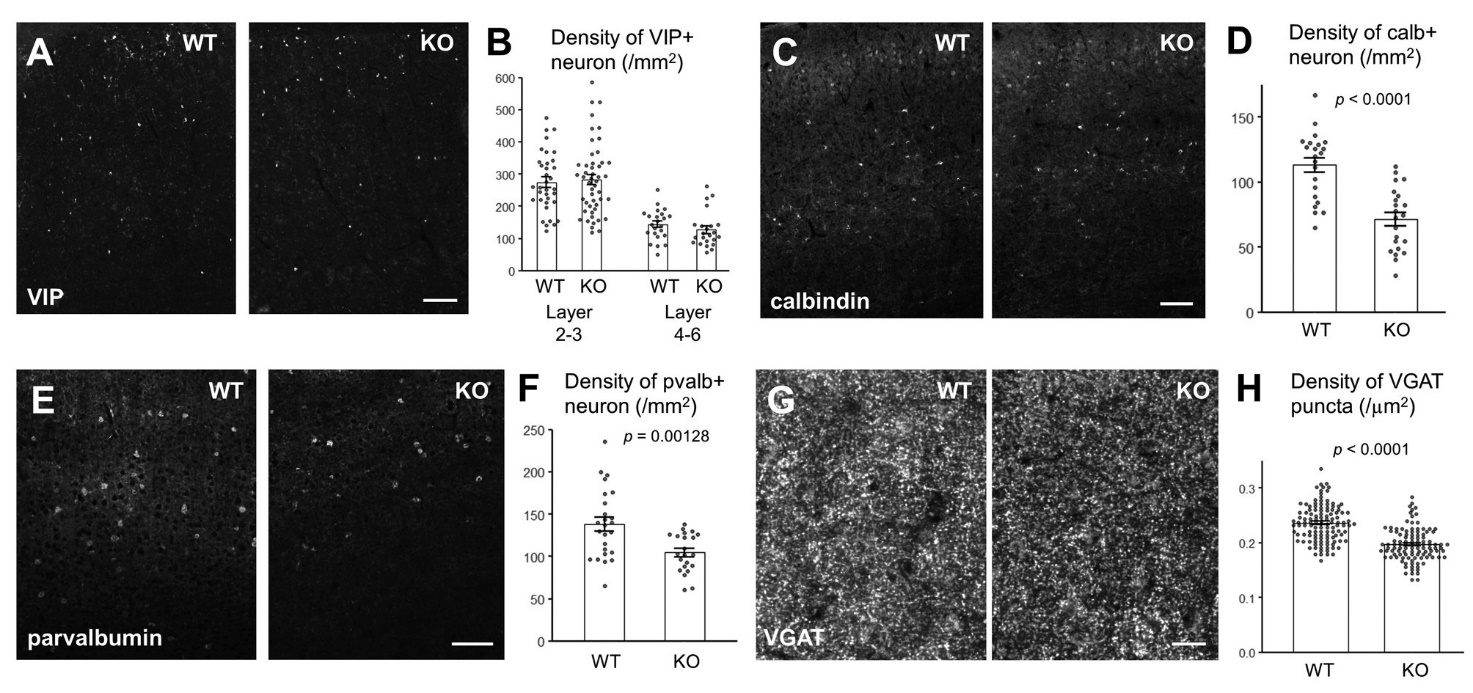
**

**Supplementary Fig. 7. Decreased density of interneurons in neonatal Fc receptor knockout mice**

**(A)** Sagittal sections of WT and FcRn KO RSC at postnatal 4 months (P4m) immunolabeled with an anti-VIP antibody. Scale bar, 100 µm. **(B)** Densities of cells positive for VIP in layers 2/3 and layers 4–6 of the RSC [5–13 images were taken per animal; WT layers 2/3 (*n* = 35); KO layers 2/3 (*n* = 49); WT layers 4–6 (*n* = 23); KO layers 4–6 (*n* = 23)]. **(C)** Sagittal sections of WT and FcRn KO RSC at P4m immunolabeled with an anti-calbindin antibody. Scale bar, 100 µm. **(D)** Density of calbindin-positive cells in layer 5 of the RSC [4–8 images were taken per animal; WT (*n* = 22); KO (*n* = 22)]. The *p*-values from Student’s *t*-test are indicated. **(E)** Sagittal sections of WT and FcRn KO RSC at P4m immunolabeled with an anti-pvalb antibody. Scale bar, 100 µm. **(F)** Density of cells positive for pvalb in layers 2–6 of the RSC [4–8 images were taken per animal; WT (*n* = 25); KO (*n* = 22)]. The *p*-values from Student’s *t*-test are indicated. **(G)** Sagittal sections of WT and FcRn KO RSC at P4m immunolabeled with an anti-VGAT antibody. Scale bar, 20 µm. **(H)** Density of puncta positive for VGAT in layers 5–6 of the RSC [24–39 images were taken per animal; WT (*n* = 120), KO (*n* = 110)]. The *p*-values from Student’s *t*-test are indicated.


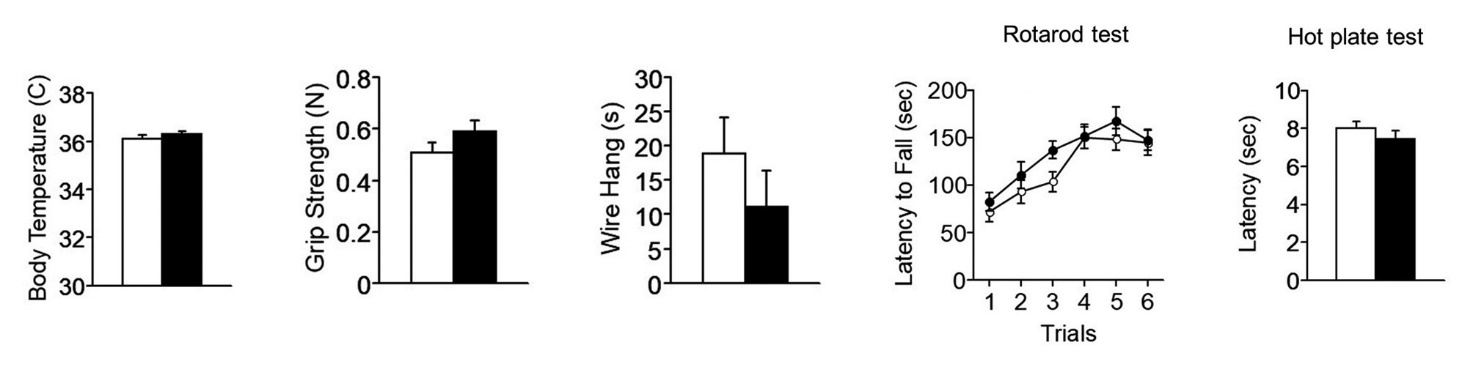


**Supplementary Fig. 8. Behavioral phenotypes of neonatal Fc receptor knockout mice**

Body temperature, grip strength, latency to fall in the wire hang test, latency to fall in the rotarod test, and latency of the first fore or hind paw response in the hot plate test in WT (open columns, *n* = 20) and FcRn KO mice (closed columns, *n* = 20).

| **Term** | ***p*-value** | **Benjamini-adjusted *p*-value** | **Genes** |
| --- | --- | --- | --- |
| cellular response to interferon-beta | 1.85E-32 | 2.77E-29 | IFI214, IFI211, OAS1A, OAS1C, IFIT1, IFIT3, OAS1G, MNDAL, GBP2, GBP4, GBP3, IFI208, GBP6, IFI209, GBP2B, GBP7, IFI204, IFI205, STAT1, TREX1, IFI203, IRGM2, IRGM1, ACOD1, IFI47, IFI202B, IIGP1, IGTP |
| cellular response to interferon-gamma | 4.52E-13 | 6.17E-11 | GBP6, GBP5, GBP2B, CCL12, GBP8, GBP7, STAT1, H2-Q7, IRGM1, ACOD1, AIF1, GBP10, CCL7, CCL5, IGTP, GBP2, GBP4, GBP3 |
| positive regulation of tumor necrosis factor production | 1.53E-09 | 1.76E-07 | H2-T23, H2-T22, H2-T10, H2-M2, H2-Q6, H2-Q7, H2-K1, OAS1A, OAS1C, H2-T-PS, OAS1G, IFIH1, TLR1, OAS2, OAS3, LGALS9 |
| positive regulation of immunoglobulin production | 4.40E-09 | 4.41E-07 | H2-T23, H2-T22, H2-T10, H2-Q6, H2-M2, H2-Q7, H2-K1, IL4RA, CD27, H2-T-PS, TNFRSF4 |
| antigen processing and presentation of exogenous peptide antigen via MHC class I | 5.69E-09 | 5.03E-07 | FCGR1, CLEC4A1, CLEC4B1, CLEC4A3, CLEC4A2, H2-K1, CLEC4A4 |
| type I interferon signaling pathway | 1.60E-08 | 1.20E-06 | IFIH1, IFITM3, OAS2, STAT1, TREX1, IRF7, OAS1A, OAS1C, OAS1G |
| cellular response to interferon-alpha | 1.81E-08 | 1.30E-06 | IFI204, IFIT3B, OAS1A, OAS1C, IFIT1, IFIT3, IFIT2, OAS1G |
| positive regulation of interferon-beta production | 4.21E-08 | 2.61E-06 | IFIH1, OAS2, OAS3, DHX58, IRF7, OAS1A, ISG15, PTPN22, OAS1C, OAS1G |
| positive regulation of antibody-dependent cellular cytotoxicity | 4.69E-08 | 2.61E-06 | H2-T23, H2-T22, H2-T10, H2-Q6, H2-M2, H2-Q7, H2-K1, H2-T-PS |
| antigen processing and presentation of exogenous peptide antigen via MHC class Ib | 6.26E-08 | 3.13E-06 | H2-T23, H2-T22, H2-T10, H2-Q6, H2-M2, H2-Q7, H2-K1, H2-T-PS |
| positive regulation of TRAIL production | 6.26E-08 | 3.13E-06 | H2-T23, H2-T22, H2-T10, H2-Q6, H2-M2, H2-Q7, H2-K1, H2-T-PS |
| inflammatory response | 8.10E-08 | 3.87E-06 | GBP5, CCL12, CXCL9, TREX1, FPR1, LY86, ACOD1, FPR3, FPR2, CXCL13, IFI202B, AIF1, SIGLECE, TLR1, C3, SERPINB1A, C4A, CCL7, IL1B, CCL5, NFKBIZ, C3AR1, TNFRSF4 |
| chemotaxis | 4.98E-07 | 1.78E-05 | RIPOR2, CCL12, CXCL9, PTGDR2, FPR1, FPR3, FPR2, CXCL13, CCL7, CCL5, C3AR1, RAC2, LGALS9 |
| positive regulation of interleukin-13 production | 1.34E-06 | 4.69E-05 | H2-T23, H2-T22, H2-T10, H2-Q6, H2-M2, H2-Q7, H2-K1, H2-T-PS |
| negative regulation of type I interferon-mediated signaling pathway | 1.93E-06 | 6.29E-05 | OAS3, TREX1, OAS1A, ISG15, OAS1C, USP18, OAS1G |

**Table S1. Gene ontology terms for the genes most altered by IgG stimulation in primary cultured microglia**

Gene ontology (GO) analysis of differentially expressed genes with IgG stimulation was performed using Database for Annotation, Visualization and Integrated Discovery functional research annotation (https://david.ncifcrf.gov). All terms were included in the category “GOTERM_BP_DIRECT, Gene Ontology Biological Process.”
